# The CONFIDENT study protocol: a randomized controlled trial comparing two methods to increase long-term care worker confidence in the COVID-19 vaccines

**DOI:** 10.1186/s12889-023-15266-x

**Published:** 2023-02-23

**Authors:** Gabrielle Stevens, Lisa C. Johnson, Catherine H. Saunders, Peter Schmidt, Ailyn Sierpe, Rachael P. Thomeer, N. Ruth Little, Matthew Cantrell, Renata W. Yen, Jacqueline A. Pogue, Timothy Holahan, Danielle C. Schubbe, Rachel C. Forcino, Branden Fillbrook, Rowena Sheppard, Celeste Wooten, Don Goldmann, A. James O’Malley, Eve Dubé, Marie-Anne Durand, Glyn Elwyn

**Affiliations:** 1 The Dartmouth Institute for Health Policy & Clinical Practice, Geisel School of Medicine, Dartmouth College, Lebanon, NH US; 2 Department of Neurology, Grossman School of Medicine, New York University, New York, NY US; 3 Department of Public Health, Brody School of Medicine, East Carolina University, Greenville, NC US; 4 National Association of Health Care Assistants, Carl Junction, MO US; 5 Department of Geriatric Medicine, University of Rochester, Rochester, NY US; 6 Long-term care worker partner, Romeo, MI US; 7 Long-term care worker partner, Maynardville, TN US; 8 Long-term care worker partner, Virginia Beach, VA US; 9 Institute for Healthcare Improvement, Boston, MA US; 10 The Dartmouth Institute for Health Policy & Clinical Practice, Department of Biomedical Data Science, Geisel School of Medicine, Dartmouth College, Lebanon, NH US; 11 Department of Anthropology, Faculty of Social Sciences, Laval University, Quebec City, QC Canada; 12 Unisanté, Centre universitaire de médecine générale et santé publique, Rue du Bugnon 44, Lausanne Switzerland; 13 CERPOP, Université de Toulouse, Inserm, Toulouse, UPS France

**Keywords:** COVID-19, COVID-19 vaccines, Vaccine confidence, Vaccine hesitancy, Long-term care workers, Intervention, Social media, Dialogue-based, Shared decision-making, Trial protocol

## Abstract

**Background:**

Clinical and real-world effectiveness data for the COVID-19 vaccines have shown that they are the best defense in preventing severe illness and death throughout the pandemic. However, in the US, some groups remain more hesitant than others about receiving COVID-19 vaccines. One important group is long-term care workers (LTCWs), especially because they risk infecting the vulnerable and clinically complex populations they serve. There is a lack of research about how best to increase vaccine confidence, especially in frontline LTCWs and healthcare staff. Our aims are to: (1) compare the impact of two interventions delivered online to enhanced usual practice on LTCW COVID-19 vaccine confidence and other pre-specified secondary outcomes, (2) determine if LTCWs’ characteristics and other factors mediate and moderate the interventions’ effect on study outcomes, and (3) explore the implementation characteristics, contexts, and processes needed to sustain a wider use of the interventions.

**Methods:**

We will conduct a three-arm randomized controlled effectiveness-implementation hybrid (type 2) trial, with randomization at the participant level. Arm 1 is a dialogue-based webinar intervention facilitated by a LTCW and a medical expert and guided by an evidence-based COVID-19 vaccine decision tool. Arm 2 is a curated social media web application intervention featuring interactive, dynamic content about COVID-19 and relevant vaccines. Arm 3 is enhanced usual practice, which directs participants to online public health information about COVID-19 vaccines. Participants will be recruited via online posts and advertisements, email invitations, and in-person visits to care settings. Trial data will be collected at four time points using online surveys. The primary outcome is COVID-19 vaccine confidence. Secondary outcomes include vaccine uptake, vaccine and booster intent for those unvaccinated, likelihood of recommending vaccination (both initial series and booster), feeling informed about the vaccines, identification of vaccine information and misinformation, and trust in COVID-19 vaccine information provided by different people and organizations. Exploration of intervention implementation will involve interviews with study participants and other stakeholders, an in-depth process evaluation, and testing during a subsequent sustainability phase.

**Discussion:**

Study findings will contribute new knowledge about how to increase COVID-19 vaccine confidence and effective informational modalities for LTCWs.

**Trial registration:**

NCT05168800 at ClinicalTrials.gov, registered December 23, 2021.

**Supplementary Information:**

The online version contains supplementary material available at 10.1186/s12889-023-15266-x.

## Background

The COVID-19 pandemic has impacted hundreds of millions of people worldwide. As of December 2022, attributable deaths exceed 6.6 million people [1]. Despite the emergence of new variants, vaccination programs continue to reduce the severity of illness, rate of hospitalizations, and overall mortality [2]. However, vaccination success depends on widespread uptake [3]. Highly infectious diseases typically require over 90% of people to be immune to protect populations [4]. Public health authorities are targeting this for COVID-19 vaccination, particularly for at-risk groups [3].

While hesitancy about vaccines existed before the COVID-19 pandemic, these concerns have been accentuated for the COVID-19 vaccines by the use of new technologies (i.e., mRNA), lack of prior data about these vaccines, limited availability of follow-up data, and provisional emergency approval [5–7]. Additionally, ideological, political, and cultural allegiances have reinforced certain concerns about vaccination [5, 6]. The internet has become a prominent source of information for many people, making it difficult to limit the spread of misinformation and disinformation while promoting evidence-based information [8–10]. This has become a major threat to confidence in the importance, safety, and effectiveness of the vaccines, which has reinforced mistrust and led to vaccine refusal [5, 8, 11].

Although long-term care (LTC) facilities in the US have been epicenters of COVID-19 outbreaks with high mortality amongst LTC residents, limited COVID-19 vaccine confidence and uptake have been reported among long-term care workers (LTCWs) [12–16]. LTCWs include people who work in LTC facilities (e.g., nursing homes, skilled nursing facilities) or home-based care. LTC positions include certified nursing assistants (CNAs) and non-clinical support, as well as traditional frontline clinical staff such as nurses, physical therapists, providers, etc. LTCWs usually serve those most vulnerable to serious complications of COVID-19 [15]. LTCWs are also more likely to be vulnerable to complications of COVID-19 themselves [15, 17, 18]. More than 50% of LTCWs involved in direct care are from minority racial and ethnic groups and socioeconomically disadvantaged groups, often working multiple jobs [17–19]. Given the vulnerability of the populations they serve, the increased risk of transmission in their communities, and their own increased risk of COVID-19 morbidity and mortality, it is important to increase COVID-19 uptake among LTCWs [14, 15].

At the time of study set up in August 2021, the US federal government announced its intent to mandate that LTCWs be vaccinated in order to be eligible to work [20]. This mandate was expanded in September 2021 to include employees in all healthcare facilities that receive federal funding [21]. While vaccine mandates logically have implications for reducing COVID-19 spread and disease severity, there are potential negative consequences as well, including possible job losses and workforce shortages if workers choose not to receive COVID-19 vaccines, and reduced workplace trust [15, 22]. This could increase tension between management and employees, decrease the number of staff to care for LTC residents, and damage the wellbeing of LTCWs 22. These effects highlight the importance of efforts to increase LTCWs’ confidence in the COVID-19 vaccines. Even for LTCWs who have been partially or fully vaccinated, increasing confidence in the vaccines will be vital for future uptake, adherence to vaccination protocols, and the acceptance of scheduled boosters.

### Rationale

The best strategy for increasing COVID-19 vaccine confidence among LTCWs remains unclear. As demonstrated by multiple reviews (and an overview of existing reviews) [23–25], addressing vaccine hesitancy is a complex task, and there is no strong evidence supporting any one single intervention. Nevertheless, there is some emerging evidence about key features of interventions that are most likely to be effective. For example, Jarrett et al. suggest that multi-component, dialogue-based interventions targeting specific unvaccinated and vaccine-hesitant populations were most effective at reducing vaccine hesitancy [25]. Other evidence indicates social media interventions can improve attitudes towards vaccines and increase uptake [26–28]. Lastly, shared decision-making (SDM) interventions that involve patients and healthcare providers sharing information and making vaccine decisions collaboratively have also been shown to improve vaccine uptake [29, 30]. Thus, in this study, we will co-develop and test two scalable, multi-component interventions targeted at LTCWs – one dialogue-based that explicitly incorporates SDM principles and a conversation aid, and the other social media-based – to improve COVID-19 vaccine confidence and other outcomes in the US LTCW population. We will compare these interventions to standard public health information on the COVID-19 vaccines available to the US population writ large.

## Methods

The trial protocol follows the SPIRIT guidelines (see Additional File 1) and CONSORT statement for cluster randomized trials [31, 32]. The three aims of our study address compelling clinical and implementation questions raised by the COVID-19 vaccines in the context of LTC.

### Aim 1

To compare the impact of two interventions delivered online: (1) a dialogue-based webinar using the existing COVID-19 Option Grid conversation aid [33] and, (2) an interactive, dynamic, and multi-component social media web application (social media website) [34], compared to enhanced usual practice (link to Centers for Disease Control and Prevention (CDC) vaccine website), on COVID-19 vaccine confidence (primary outcome) and other secondary outcomes among LTCWs.

#### Hypothesis 1.1

Each intervention will be superior to enhanced usual practice at increasing vaccine confidence.

#### Hypothesis 1.2

The dialogue-based webinar intervention will be superior to the social media intervention at increasing vaccine confidence.

We predict superiority of the dialogue-based webinar intervention based on stronger evidence that exists for dialogue-based approaches and SDM for improving vaccine hesitancy and/or uptake, as compared to social media approaches. While our social media website intervention includes elements of dialogue, this component is more explicit in the webinar intervention (see ‘Interventions and control’).

### Aim 2

To determine if LTCWs’ characteristics and other factors mediate and moderate the interventions’ impact on vaccine confidence and other secondary outcomes.

#### Hypothesis 2.1

Increased perceptions of feeling informed about the vaccines, identification of vaccine information and misinformation, and trust in vaccine information provided by different sources will explain (mediate) the relationship between the interventions and vaccine confidence, as well as other secondary outcomes.

#### Exploratory

We will conduct exploratory heterogeneity of treatment effects (HTE) analyses to identify whether certain participant characteristics and beliefs moderate the relationships among each of the interventions and vaccine confidence, as well as other secondary outcomes. Variables to be explored will include, but are not limited to, vaccination status, religious beliefs, perceived influence of others, age, race, ethnicity, and personal experiences with COVID-19.

### Aim 3

To explore the implementation characteristics, contexts, and processes needed to sustain and scale up the use of interventions designed to increase vaccine confidence among LTCWs.

#### Hypothesis 3.1

Co-developing and adapting the implementation strategy and outcomes with LTCWs and other stakeholders will facilitate implementation.

### Study design

**Fig. 1:**
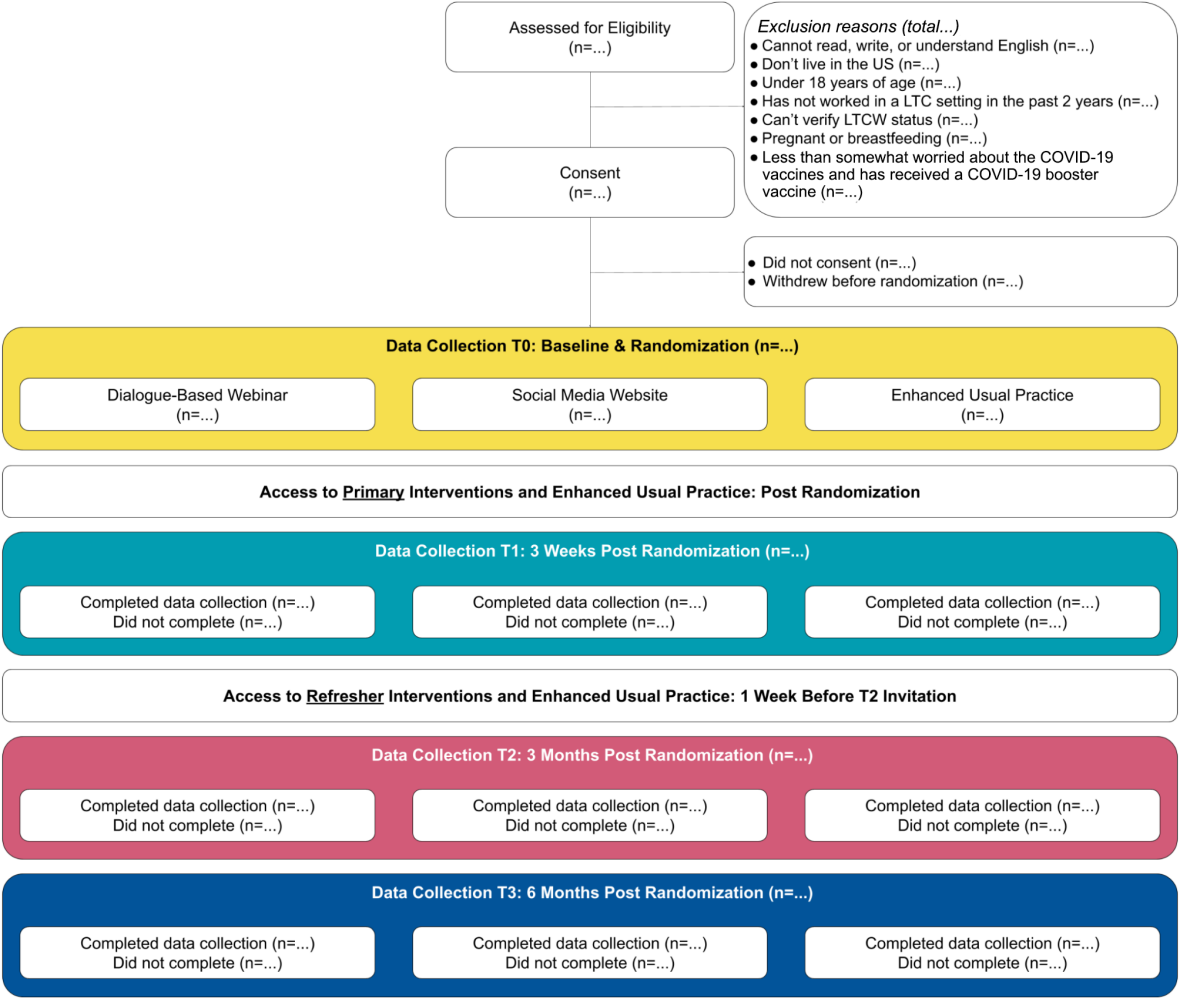
CONSORT diagram Note: Participants must complete T0 to be enrolled in the study. Access to and completion of T2 and T3 follow-up surveys are not dependent on completion of a prior follow-up survey

We will conduct a randomized controlled trial (RCT) with three arms and a hybrid-effectiveness implementation design type 2 [35] (Fig. 1). This design includes evaluating effectiveness in the randomized trial alongside an exploration of implementation (tasks 1–5 of implementation mapping) [36]. Contingent on the trial results, implementation will then be evaluated in a sustainability phase after randomized trial assessments are completed (see ‘Aim 3’). The recruitment goal for the RCT is 1,800 participants (600 per arm), and randomization will be at the individual person level.

We will draw on elements of Community Based Participatory Research (CBPR), and co-design and co-manage this study with LTCWs, the National Association of Health Care Assistants (NAHCA), and the Institute for Healthcare Improvement (IHI). Trial data will be obtained primarily via four self-reported participant surveys, delivered online over a period of approximately six months (Fig. 1). In the trial set-up period, an additional online panel survey was administered to a sample of 592 people from the general population who were demographically representative of LTCWs. This survey tested several novel and adapted trial screening and outcome questions and contributed to intervention design [34] (survey details available on request).

### Setting

We will use a combination of online and in-person recruitment strategies in the US. In-person recruitment will occur at a convenience sample of LTC settings in North Carolina, identified by our collaborator (NRL), who has extensive professional LTC experience and trusted relationships with LTC leaders in the North Carolina area. Settings will include large and smaller size for-profit and not-for-profit nursing homes and continuing care retirement communities in both rural and urban locations. Additional settings may include Virginia and the New England region.

### Participants

People will be eligible to join the trial if they self-identify as: (1) 18 years of age or older, (2) living in the US, (3) having worked in a LTC setting in the past two years, (4) able to verify their LTCW status, (5) not currently pregnant or breastfeeding, (6) able to read, write, and understand English, and (7) being at least somewhat worried about the COVID-19 vaccines and/or not having received any COVID-19 booster vaccine (also includes those who have not completed a primary COVID-19 vaccine series).

Eligible LTC settings will include nursing homes, skilled nursing facilities, assisted living facilities, home health care, hospice care, and retirement communities. The two-year timeframe will avoid excluding those who are currently unemployed, and will approximately cover the duration of time since the COVID-19 pandemic began. To ensure a high proportion of study participants represent our low confidence target group, we will use two criteria – worry about the vaccines and booster uptake – to screen participants for eligibility. These criteria are based on a preliminary analysis of panel survey data, which found associations between booster intent, worry about the vaccines, and vaccine confidence.

## Interventions and control arm

### Theoretical and practical foundations

**Fig. 2:**
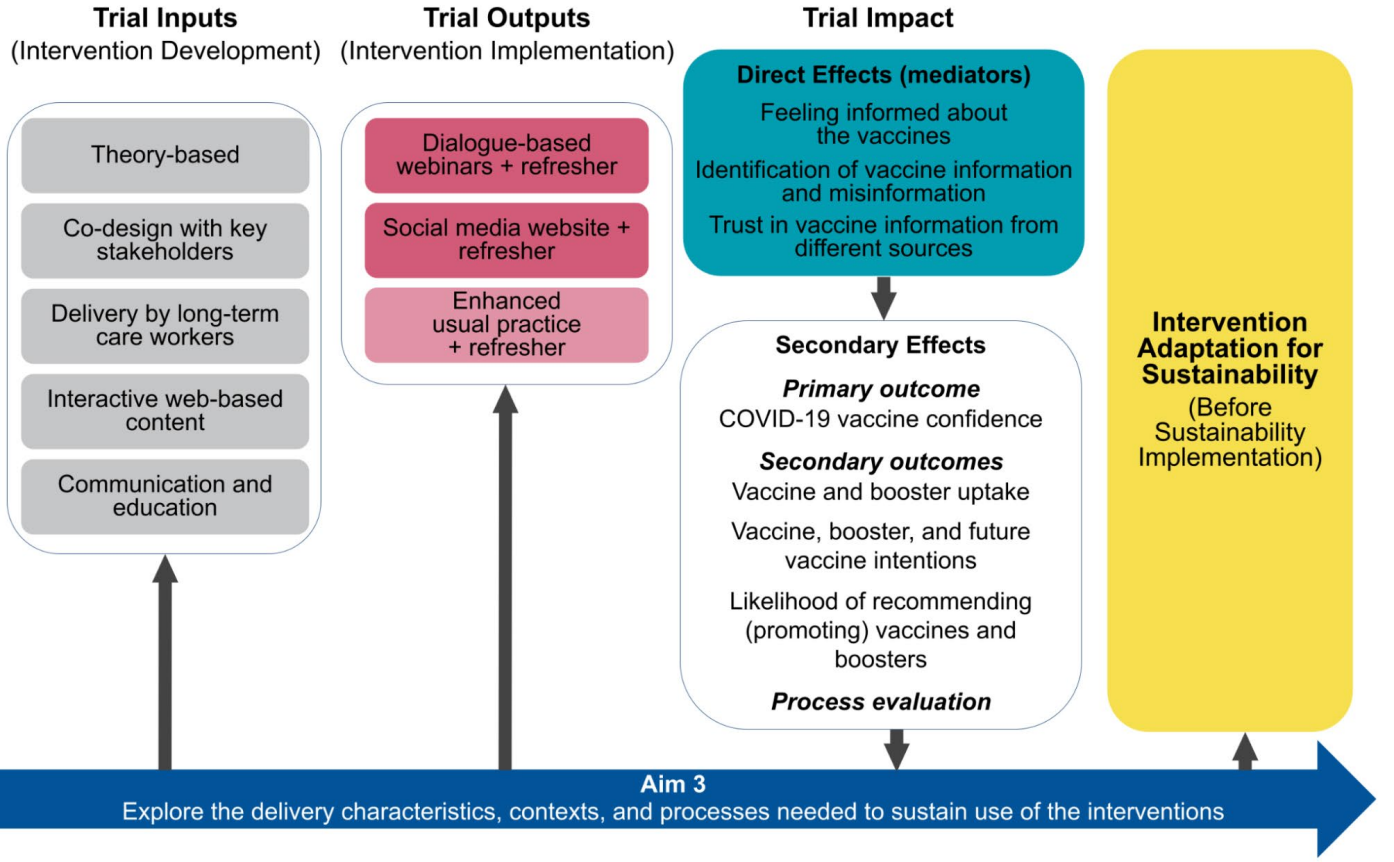
Randomized controlled trial logic model

We designed two multi-component, interactive, online interventions (arms 1 and 2), informed by theoretical and practical information from the vaccine confidence and hesitancy literature. Notably, our intervention development and hypothesized mediators (see Fig. 2) were initially informed by Peretti-Watel’s vaccine hesitancy framework, which conceptualizes vaccine decision-making as a process. The framework also distinguishes between two types of vaccine hesitant people: (1) those with poor knowledge of and/or indifference to vaccination issues, and (2) those who are interested in vaccination issues and seek more information, yet are hesitant [37]. We were also informed by the Report of the SAGE Working Group on Vaccine Hesitancy and its conceptualization of the “three Cs” of vaccine hesitancy: Confidence, Complacency and Convenience; and, we considered all three Cs [38]. Additionally, our interventions were informed by the literature on reducing vaccine hesitancy, which suggests multi-component, dialogue-based interventions (such as conversation-based and social media interventions) are most effective, as well as those tailored for specific populations [25]. The interventions were co-designed, adapted, and pre-tested with LTCWs as well as NAHCA, IHI, and other co-investigators. The control arm (enhanced usual practice) is online COVID-19 vaccine information provided by the CDC.

### Development

The overall designs of the dialogue-based webinar and social media website interventions were informed by prior studies [25, 26, 29] and developed using participatory research approaches [39, 40]. In developing content for each intervention, our goal was to maximize consistency of major topics across interventions, in order to isolate effects of each intervention delivery method.

To inform the intervention content, we initially derived topics from several sources, including social media monitoring, news surveillance, online public opinion polls [41, 42], and peer-reviewed literature [38]. The major topics for inclusion in both interventions were then refined via interviews with our LTCW partners as part of Aim 3. Additional information was gathered to inform the development of the social media website intervention, the details of which are published elsewhere [34]. The final list of major topics includes COVID-19 in general, vaccine benefits, vaccine risks, and vaccine development [34].

We also developed community standards to ensure all participants felt welcome and comfortable when engaging with the interventions. These standards, which are similar across both interventions, were based on the rules of existing vaccine discussion forums and were co-created with our LTCW partners [34]. Also included in both interventions is a video that we developed featuring LTC residents voicing their views on why it is important for LTCWs to be vaccinated.

In response to low intervention engagement in one trial arm (webinar attendance) experienced in the first several weeks of data collection (see progress of research in Additional File 6), we developed intervention ‘refreshers’ for each trial arm. The refreshers for trial arms 1 and 2 are briefer, modified versions of each primary intervention. They include content on new and emerging COVID-19 and vaccine-related topics, as well as popular questions trial participants have voiced through their primary intervention engagement. New and emerging topics were derived from several sources, including social media monitoring, news surveillance, online public opinion polls [43], and consultation with our LTCW partners and other stakeholders. Topics for inclusion were refined primarily via a poll to identify stakeholders’ information needs (process adapted from [44]). Refreshers for trial arms 1 and 2 will be updated as required when there is sufficient new and emerging COVID-19 related public discourse.

### Intervention 1: dialogue-based webinar

#### Primary intervention

We will invite LTCWs to attend one scheduled webinar (online video-based discussion session). The webinars will be hosted in the Zoom platform, will run in groups of no more than 20 participants, and will occur at various times to accommodate different schedules. Each webinar will be led by a LTCW peer trained in SDM principles. It will also be co-facilitated by a physician with expertise in COVID-19 vaccination, and a communication expert with experience in SDM.

The webinar agenda (see Additional File 2) starts with a LTCW facilitator introducing the session and going over general instructions. Next, the physician facilitator reviews the COVID-19 vaccine Option Grid™ conversation aid and other related topics of interest using the principles of the SDM three-talk model [45]. A link to the online Option Grid™ is also sent to participants prior to their webinar commencing. Participants are then presented with the four major content topics pertaining to COVID-19 and the vaccines (consistent with those listed above in ‘Development’) that are also sent to participants prior to their webinar commencing. They are then asked to vote (via a Zoom poll) on the two topics they are most interested in discussing. The physician facilitator then answers questions from participants (asked verbally or via the text chat) related to the top voted topics. The webinar ends with the LTC resident video described earlier. Throughout the webinar, the communication expert assists with any discussions where needed, responds to study-related questions/issues, and helps with technical troubleshooting. Webinar durations will vary – running up to 1 hour, or less if there are no remaining questions.

### ***Refresher intervention***

The dialogue-based webinar refresher will be emailed to all participants in this arm one week prior to their T2 survey invitation (see Fig. 1; Additional File 3). The refresher is a pre-recorded webinar with real facilitators and mock participants, lasting approximately 20 min. The structure of the recorded webinar will closely resemble that of the primary intervention, and will include a review of the COVID-19 Option Grid™, question and answer discussion, and LTC resident video. It will be available to participants as a video and an audio-only recording.

### Intervention 2: social media website

#### Primary intervention

We will invite LTCWs to visit a COVID-19 social media website. The website is curated with popular and topical posts from social media platforms such as Facebook, TikTok, and YouTube that are made by medical experts, LTCWs, and other creators. The content and topics addressed on the website are dynamic in nature, with two new posts added daily.

The top of the website homepage features the four major content topics pertaining to COVID-19 and the vaccines (consistent with those listed above in ‘Development’). Participants can navigate the website by selecting from these major topics, selecting subtopics listed in the sidebar menu or as hashtags assigned to each post, or by scrolling the remainder of the homepage (organized as an infinite scroll of all posts, sorted by most recently uploaded). The website supports participant interaction with features such as reactions and comments. Participants also receive email notifications when other users react or reply to comments they have made.

Three special website users affiliated with the study team who have worked as CNAs in LTC settings contribute to the website as ‘Community Ambassadors’. Their role is to promote user engagement by reacting and replying to posts and participants’ comments based on their own experiences as CNAs. Ambassadors also provide factual information on COVID-19, the vaccines, and boosters should participants ask any direct questions.

More on the development of the social site and its mechanics is detailed elsewhere [34].

### ***Refresher intervention***

The social media website refresher will be sent to all participants assigned to this arm one week prior to their T2 survey invitation (see Fig. 1; Additional File 3). The refresher is an email newsletter featuring a small selection of website content presented as linked thumbnails. Participants can click on a thumbnail to be directed to the social media website.

### Control arm: enhanced usual practice

#### Primary control

We will invite LTCWs to view COVID-19 vaccine information on the CDC website [46]. The CDC website addresses common questions about the COVID-19 vaccines and provides information on related topics, such as specifics about getting vaccinated and staying up to date on the vaccines and boosters. To address potential concerns about intervention inferiority, LTCWs in the enhanced usual practice arm will be given access to both interventions after the 6-month follow-up for all participants is completed. We will then monitor vaccine confidence again in this arm.

### ***Refresher control***

The enhanced usual practice refresher will be sent to all participants assigned to this arm one week prior to their T2 survey invitation (see Fig. 1; Additional File 3). The refresher is an email featuring the same CDC website link initially sent after trial arm allocation.

### Allocation to interventions

Participants will be enrolled online via the Qualtrics survey platform [47]. We will use the Qualtrics randomizer function to allocate participants to a trial arm. At the end of the T0 (baseline) Survey, Qualtrics will automatically assign participants’ trial arm using a 1:1:1 allocation ratio. As randomization will occur electronically at the time of baseline survey completion, the sequence is concealed to participants at the moment of randomization.

### Changes to intervention allocation

There are no established criteria for discontinuing or changing the intervention each participant is allocated to, due to the low risk nature of the trial. We will, however, minimize a participant’s ability to interact with their intervention (e.g., via muting/blocking) in the event that they breach community guidelines.

### Blinding

Due to the nature of the interventions and intervention delivery, it is not possible to blind participants, facilitators, or research team members to the trial arm participants are assigned. Participants may be aware that they are in either a control or intervention arm. Additionally, there will be separate, non-overlapping groups of research team members and stakeholders responsible for the delivery of each intervention. The data analyst (PS) will be blinded to arm allocation where possible.

## Procedures and data collection

### Recruitment

Our recruitment strategy (see Table 1) will be guided by our LTCW stakeholders, including our NAHCA and East Carolina University (ECU) collaborators. Our stakeholders initially recommended online recruitment given its potential reach and practicality with the continued circulation of the COVID-19 virus. We will also recruit via a convenience sample of LTC settings identified by our study collaborators and stakeholders. Recruitment messaging (i.e., emails, social media ads, posters, table tents, and business cards) will include links to a study recruitment website and/or study eligibility screening questions. The recruitment website will include brief, plain-language study information in both video and written formats, and will contain links to access the study screening questions.

**Table 1:** Summary of LTCW recruitment methods

Method	Details
**In-person**	
Visits to LTC settings	Information sessions to promote the study led by a study collaborator or team member
Conferences	Study materials and messaging distributed via stakeholders
LTC setting outreach	Study materials sent to the facility for display and distribution
**Online**	
Listserv emails	Sent to all NAHCA members by our stakeholder partner, NAHCA
Social media paid advertisements	Paid ads on Facebook and Instagram distributed by NAHCA
Social media posts	Messages shared in Facebook groups/pages owned or known by our stakeholder partner, NAHCA

### Consent and enrollment

Those interested in joining the study will first be screened for eligibility at the beginning of their baseline survey. If they meet all eligibility criteria, they will proceed to a study information sheet providing similar information to a traditional consent form (available on request). To improve accessibility and understanding of the study information, we developed an animated video version of the information sheet. Participants will be able to choose if they want to watch the video, read the information sheet, or both. The content will be identical. Participants will then indicate that they understand the information they just read or watched, and consent via an electronic checkbox to proceed to the remainder of the baseline survey.

### Baseline data collection

In addition to screening and consent, the baseline survey will collect participants’ LTCW verification information, contact details, preferred method of contact (email or text), how they heard about the study, participant characteristics, contextual information, and baseline study outcome data (see ‘Outcomes’).

### Verification process

Because of the online nature of the trial, incentives provided, and early instances of fraudulent activity (see progress of research in Additional File 6), we will implement several strategies to prevent, detect, and respond to fraudulent study enrollment. The strategies we will adopt were informed by a prior review of methods [48] and recommendations [49, 50] on this topic. We also engaged our stakeholders in this development process to ensure a range of strategies feasible and acceptable to LTCWs.

First, in the baseline survey, we will incorporate a Qualtrics reCAPTCHA bot detection filter [48, 49, 51], cookie-based settings that prevent multiple submissions from the same web browser [48, 49, 52], and a message discouraging duplicate survey completion [48, 49]. Second, we will collect information from participants that will allow us to verify their identity and that they have worked in a LTC setting in the past two years.

After providing consent, participants will be asked to provide the name of their current or former LTC workplace, the type of LTC setting, their role, and the length of time that they have worked in LTC. Participants will then choose one of four different options for LTCW verification, as outlined in Table 2. We will use the information provided in Table 2 and triangulate it with other information provided by participants (e.g., workplace information) to verify LTCW status. Reminder messages (text and email) and, ultimately, phone conversations and Zoom calls [48, 49], will be used to gather further information from participants who fail to provide the requested LTCW verification information, or where further clarification on information provided is needed.

**Table 2:** Methods of verifying long-term care worker status

Verification method	Details
Work ID badge	Upload a photo of their work ID badge, or show us their badge over video call.
Recent pay stub	Upload a photo of a prior pay stub from within the past two years, or show us their pay stub over a video call.
Certification or professional license number	Provide their Certification Number or Professional License Number, the state they are registered in, and their associated professional role (e.g., certified nursing assistants in most states, health care providers). Numbers are then confirmed via public registry checks.
Email from work account	Email us from their workplace email account with their first and last name, the name of their workplace, and the name of the study.

We will also employ TransUnion’s TLOxp verification service (www.tlo.com), to confirm the identity of participants recruited online [50]. TLOxp aggregates publicly available databases and records to authenticate and verify identity information. We will develop a standardized process for identity verification using participant name, zip code, age range, cell phone number, and email address.

Participants who do not pass a verification check (LTCW status and/or identity) will be given the opportunity to provide additional information. If they do not provide verifiable information, they will be sent a message stating they have been unenrolled in the study [49]. Due to a delay in implementing TLOxp identity checks (see Additional File 6), some of the identity checks will be retrospective (i.e., post study completion). Those who cannot be verified will be removed from the study sample prior to statistical analyses (see Additional File 4).

Participants recruited in person will be presented with the same verification survey questions. However, due to verification not being necessary for this subpopulation, we will primarily rely on their reported workplace name to confirm their legitimacy as LTCWs. Identity checks will also not be performed on this group.

### Intervention delivery

#### Primary interventions and control

Immediately following trial arm allocation, participants will be presented with a brief description of their assigned trial arm. They will be told it is important they engage in their relevant trial arm activity within the next three weeks. Upon submitting their baseline survey, participants will be automatically redirected to another website based on their assigned arm. Participants in the dialogue-based webinar arm will be able to register for an upcoming webinar at a pre-specified date and time via a separate Qualtrics survey and Zoom registration page. Those in the social media website arm will be able to create user accounts and access the site immediately. Those in the enhanced usual practice arm will have direct access to the public CDC website.

Participants will receive study enrollment confirmation messages immediately after joining and several additional reminders pertaining to their assigned trial arm over the following two weeks (see Additional File 3). We will use a combination of email, text, and phone calls for these reminders, with most messages sent to the preferred method of contact (text or email) that the participant selected in their baseline survey.

#### Refresher interventions and control

Participants will be given access to their respective refresher intervention or information via email one week before receiving their T2 Survey invitation (see Fig. 1 and Additional File 3). They will receive one reminder email two days later featuring the same content.

### Follow-up data collection

Participants will be invited to complete three follow-up surveys. The surveys will be sent three weeks post-baseline survey/randomization (T1), three months post-baseline survey (T2), and six months post-baseline survey (T3). Participants will be sent a pre-reminder before each survey invitation is sent, and up to four reminders afterwards for surveys not completed. We will use a combination of email, text, and phone calls for these reminders, with most messages sent to participants’ preferred method of contact. If a follow-up survey is not completed, participants will still be sent subsequent surveys.

We will not collect data on the reasons people decline consent, drop out before randomization, withdraw from the study, or do not complete one or more follow-up surveys. We will however seek information on reasons people did not engage with primary interventions and refreshers (see ‘Outcomes, Process evaluation’ and ‘Aim 3’).

### Retaining participants

We will maximize retention by: (1) using short-form questions and measures when possible; (2) using automated reminders and multiple methods of communication, and (3) compensating participants with a $30 gift card for each survey completed. Brueton et al. identified monetary incentives as an effective way of improving participant retention [53].

### Outcomes

We will collect primary and secondary outcomes across four online surveys that we tested with our LTCW partners. Sample surveys are available on request. We will also collect online activity data from both interventions. Timepoints for primary and secondary outcome assessment are specified in Additional File 4.

### Primary outcome measure

Our primary outcome is COVID-19 vaccine confidence, which will be assessed using an adapted version of the Vaccine Confidence Index (VCI) [54]. The VCI measures confidence in vaccine importance, safety, and efficacy. Each item will be rated on a 5-point scale ranging from 1 (‘strongly disagree’) to 5 (‘strongly agree’). Participants are considered ‘confident’ (score of ‘1’) if they respond ‘agree’ or ‘strongly agree’ on all three items. This scoring approach was determined via panel survey data, in which we explored optimal thresholds that predicted vaccine uptake.

### Secondary outcome measures

***COVID-19 vaccine uptake and intent.*** We will assess uptake of the COVID-19 vaccines (any dose, initial series completion, and booster completion) using four questions that we developed. For those who report not being vaccinated or boosted, intent to get a COVID-19 vaccine (initial series or booster) will be assessed using two questions broadly adapted from prior work [55]. All participants will also be asked if they would get regular vaccines in the future if they are recommended, using a single question.

***Likelihood of recommending (promoting) COVID-19 vaccination.*** Adapted Net Promoter Score (NPS) questions [56] will assess the likelihood that participants would recommend (1) COVID-19 vaccination to others who are unvaccinated, and (2) COVID-19 booster vaccination to a coworker. Similar questions have been recommended previously [57, 58]. We will adopt the traditional scoring approach that categorizes respondents as promoters, passives, or detractors.

***Feeling informed about the COVID-19 vaccines.*** We will assess the degree to which participants feel informed about the COVID-19 vaccines (have enough information and understand that information) using two questions that we developed. Our operational definition of feeling informed was influenced by the Decision Self-Efficacy Scale [59].

***Identification of COVID-19 vaccine information and misinformation.*** We will assess identification of COVID-19 vaccine-related information and misinformation using four questions that were shown to have low rates of correct identification in our preliminary pilot work. We developed two questions and two were adapted from prior work [42].

***Trust in COVID-19 information from different sources.*** We will assess trust in COVID-19 information provided by different people and organizations using three items broadly adapted from prior work [60].

### Other data collected

***Participant characteristics.*** We will assess participant characteristics using a combination of existing, adapted, and self-developed questions for age, gender [61, 62], zip code, educational attainment [63], race and ethnicity [63], health insurance [63, 64], health literacy [65, 66], religiosity [67], LTCW role, LTC setting type, duration of experience in LTC, extent of others’ influence in COVID-19 vaccination, and baseline vaccination status.

***Contextual factors.*** We will identify contextual factors that may contribute to COVID-19 vaccine intentions and decisions, including personal COVID-19 and vaccine experiences and participation in other COVID-19 vaccine research, using a single question that we developed.

***External factors.*** Outside of the study surveys and throughout the trial, we will monitor external factors that may impact participants’ views and actions towards the COVID-19 vaccines. This may include policy and vaccine mandate changes for LTCWs and changes in the nature of the pandemic, among other things.

***Intervention engagement.*** We will monitor the extent to which participants engage with their assigned primary and refresher intervention content. We will collect online activity data (i.e., social media website user history, webinar attendance records, email click rates) and participant self-reported engagement data via surveys. We will prioritize the use of online activity data to minimize potential measurement error [68]. However, survey questions will be used where online activity data is not available or is incomplete; for example, to determine engagement with the webinar refresher recording (adapted from [68]) and enhanced usual practice information. Data on engagement will be used to inform secondary trial analyses, as well as Aim 3.

***Process evaluation.*** We will conduct a process evaluation as a component of Aim 3 to inform implementation and sustainability activities. Process evaluation questions will be administered in all follow-up surveys (T1-T3). Acceptability of the interventions and control arm will be determined via adapted NPS questions [56] (i.e., likelihood of recommending to a coworker). Similar approaches have previously been used for evaluating SDM interventions [68–71]. We will also assess how new the information was that participants were exposed to, the comprehension of and trust in the information (informed by [72]), the degree to which they felt listened to and respected by those running the interventions (informed by [73]), and reasons for not engaging with the primary or refresher interventions or control arm (adapted from [71]).

### Sample size and power calculation

Using historical VCI data [74] and current vaccination rates [75], a binary classifier for vaccine confidence was identified based on VCI responses across all three questions, with ‘confidence’ defined as responding ‘agree’ or ‘strongly agree.’ This binary classifier was significantly correlated with rates of vaccination (p = 0.0001). The sample size of 1,800 LTCWs (600 per arm) provides 80% power to detect an 8% difference in the rate of ‘confident’ participants in each of the three different pairwise comparisons of study arms at family-wise type I error rate of 0.05. The sample size is sufficient to retain 80% power to detect a 10% difference (assuming outcomes are randomly distributed across retained and lost participants) after 40% attrition.

### Statistical analysis

All analyses pertaining to study Aims 1 and 2 will be conducted on an intention-to-treat (ITT) basis (i.e., the arm participants were assigned to) and as-treated basis (i.e., whether they engaged with their assigned intervention or control). A detailed data analysis methodology, including planned statistical tests, timepoints for outcome assessment, and treatment of missing data, is included in Additional File 4.

For Aim 1, hypothesis 1.1, we will conduct one-tailed tests (superiority analysis) to compare the impact of each of the two intervention arms against the enhanced usual practice arm on primary and secondary outcomes. For Aim 1, hypothesis 1.2, we will conduct a two-tailed test (equivalence analysis) of primary and secondary outcomes between the two intervention arms. While we hypothesize that the dialogue-based webinar intervention (Arm 1) will be superior to the social media website intervention (Arm 2), a finding of superiority, inferiority, or no distinguishable difference will be a valuable finding.

For Aim 2, hypothesis 2.1, our mediation analyses seek to identify the relationship between the interventions, mediator variables, and primary and secondary outcomes. We are interested in whether interventions operate through the mediator rather than directly affecting the outcome. If the results for Aim 1 are non-significant, we will determine whether it is a null effect of the intervention on the mediator or a null effect of the mediator on the outcome. We will determine mediation strength and mechanism generalizability by comparing effects across subgroups.

For Aim 2, our exploratory moderation analyses (also referred to as heterogeneity of treatment effects (HTE) analyses) seek to understand whether certain participant characteristics and beliefs influence the relationship between the interventions and vaccine confidence, as well as other secondary outcomes. We will explore moderators including (but not limited to) vaccination status, religious beliefs, age, race, ethnicity, perceived influence of others, and personal experiences with COVID-19.

Because of the size, scope, and complexity of this study, exploratory analyses of relationships within the data will be conducted to identify factors to inform future analysis (see Additional File 4). Exploratory, hypothesis-free analyses will be performed using data clustering to analyze participants’ demographic, geographic, or temporal links, which may define statistically unlikely outcome groupings.

### Aim 3

We will examine the implementation and sustainability potential of the dialogue-based webinar and social media website interventions using an implementation mapping approach informed by relevant domains of the Consolidated Framework for Implementation Research (CFIR) [36, 76, 77] (see Fig. 2 and Additional File 5 for Aim 3 planned activities). Aim 3 will also include a separate but related process evaluation component.

We will interview a purposive sample of stakeholders in the planning, trial, and sustainability phases of the study, including LTCW partners, LTC leaders, trial investigators, partner organizations, and participants in the two intervention arms (n = up to 100 total based on data saturation within predetermined subgroups). These interviews will explore the delivery characteristics, contexts, and processes needed to sustain LTCWs’ use of the interventions. Trial participants will receive a $30 gift card for participating in an interview. We will also collect online activity data from both interventions, field notes, and observations of the adaptations made to the interventions during the trial using FRAME (expanded framework for reporting adaptations and modifications to evidence-based interventions) [78]. After completion of the RCT, intervention adaptations will be made (also tracked via FRAME) based on trial results and informed by Aim 3 interviews with key stakeholders. In the sustainability phase, we will open access to adapted interventions to all LTCWs outside the study participant sample and identify engagement rates via available online activity data. The Aim 3 process evaluation encompasses fidelity measurement, dose, reach, and reactions to the interventions and enhanced usual practice information (see ‘Outcomes, Process evaluation’).

### Data management

Oversight of trial data will be the responsibility of the Program Director, Trial Manager, and Data Manager. Prior to trial recruitment commencing, we will work with a third party vendor to implement a customized Salesforce platform for participant management throughout the trial. This will include setting up integrations with Qualtrics, Zoom, Mogli (text message vendor), and ActiveCampaign (email distribution program). Using Salesforce will facilitate the automated distribution of emails and text messages to participants, and the continued management of participants at the group and individual level, throughout the trial.

Any identifying data exported from Qualtrics, Salesforce, the social media website, Zoom, or ActiveCampaign will be stored on computers, external hard drives, and cloud-based platforms that are password-protected. Access to all software and platforms where identifying data are stored or handled will only be given to research team members involved in data collection, management, and/or analysis and reporting, on a need-to-know basis.

### Trial management and monitoring

### Monitoring enrollment

Trial enrollment will be monitored weekly using Salesforce. The number of people who are ineligible and screened out and corresponding reasons why will also be reviewed. We will periodically monitor certain demographic characteristics (gender, age, race/ethnicity, education) of our study sample to determine if they are representative of the national LTCW population [19]. Where possible, we will focus subsequent enrollment on any underrepresented groups.

### Adherence to protocol

We will train all relevant co-investigators, research staff, intervention facilitators, and other stakeholders in delivering the interventions and facilitating recruitment, verification, and retention according to the procedures outlined in the protocol. The protocol will be available to all team members and key stakeholders. Standard Operating Procedures (SOPs) will also document detailed tasks to be routinely performed by research staff to enhance fidelity and consistency of study functions.

Activity within each intervention will be monitored by research staff to ensure fidelity of intervention delivery and participants’ adherence to community standards. Fidelity observation grids will be completed weekly. Feedback on protocol adherence or nonadherence will be shared with relevant parties involved in intervention delivery on a regular basis.

### Trial management

Dartmouth College will be responsible for centralized trial management and general oversight. The Dartmouth research team will maintain all aspects of the trial and will work closely with each collaborator (NAHCA, ECU, IHI) to coordinate all trial activities. The Center for Program Design and Evaluation (CPDE), a research service center at Dartmouth, will conduct the process evaluation as part of Aim 3. The research team will meet with CDPE on a regular basis to ensure coordination, access to Aim 3 interviews and necessary intervention data and information, and optimal timing of process evaluation activities.

### Advisory groups

Three advisory groups will be assembled and will meet regularly throughout the study. A Stakeholder Advisory Group (SAG) will review progress towards study goals and objectives and offer feedback and guidance throughout. The SAG will consist of ten LTCW partners and other key stakeholders (members from advocacy, policy, and LTC organizations) and will meet on a quarterly basis. A Trial Steering Group (TSG) consisting of co-investigators, four core LTCW partners, and expert consultants in vaccine confidence, will monitor trial progress, offer advice, and make final decisions on pending study questions. The TSG will also meet quarterly. The group of ten LTCW partners will also meet bimonthly to discuss study progress and to offer feedback and advice on study materials, plans, and progress.

### Data Safety and Monitoring Board

A Data Safety and Monitoring Board (DSMB) will be convened to provide additional oversight of the trial and to assess data safety, and will serve in an advisory role in making recommendations to the study staff. The DSMB will consist of four members (inclusive of the chair), including experts in or representatives from the areas of data safety monitoring, statistics, and clinical trial methodology. The DSMB will operate independently from the study funder and research team. It will meet three times during the course of the trial. The DSMB will review the protocol, data collected, and the performance of study operations and other relevant issues.

Trial activity will also be monitored by members of the study team on an ongoing basis. We believe the likelihood of serious adverse events in the trial to be extremely low, as there are no invasive procedures related to the interventions. All potential adverse events or unanticipated events/problems will be reported to the Principal Investigators (PIs). All events will be discussed by the PIs and relevant members of the study team. Any event deemed to be reportable will be submitted to Dartmouth Committee for the Protection of Human Subjects (CPHS), and if necessary, the DSMB will convene urgently to review the event in question and advise the PIs on any risk mitigation plans.

### Ethics and dissemination

This study was approved by the Dartmouth CPHS [STUDY00032340]. Protocol modifications will be reported to the Dartmouth CPHS, DSMB, and/or the study funder, where relevant and required.

Once all study data are collected and analyzed, we will develop a pictorial lay summary of the research aims, methods, and key findings. The summary and the final results publication will be sent to interested participants. In addition, we will engage in broader dissemination efforts targeting community stakeholder organizations (e.g., press releases, newsletters, social media outreach). Determination of authorship on all results publications will adhere to the International Committee of Medical Journal Editors authorship criteria.

We will also prepare a full data package of anonymized RCT data. This data package will be maintained for at least seven years. As per funder requirements, we will make the package available to a data repository (designated by the funder) to facilitate data sharing with the broader scientific community.

### Progress of research

Planning and executing a trial during the COVID-19 pandemic has presented unique challenges to the research process, requiring several unanticipated protocol adaptations as the pandemic evolved. We have also experienced increasingly sophisticated instances of fraudulent enrollment activity, necessitating continued adaptations to our verification processes. We have detailed these changes and their justifications in Additional File 6.

## Discussion

Our LTCW partners and other relevant stakeholders have highlighted the critical importance of improving confidence in the COVID-19 vaccines and boosters and implementing effective interventions quickly. The hybrid design we have chosen targets actionable findings and translational gains. As far as can be determined, this study is the first to use a participatory approach in developing, evaluating, and implementing interventions to improve vaccine confidence in LTCWs.

We have planned and will conduct this complex trial in the context of an evolving pandemic. Changes in the circulation and severity of COVID-19, implementation and subsequent relaxation of vaccine mandates, and various policies and restrictions in LTC settings may impact LTCWs’ interest in participating in the study over the course of the trial. These changes may also play a role in how participants respond to and are impacted by the interventions, which we will explore in our planned statistical analyses.

We anticipate that this research will contribute to understanding and implementing the best ways to reach a target population that has historically been understudied. Our findings will be highly relevant to public health officials and policy makers as they explore ways to improve vaccine confidence among LTCWs who care for a very frail and at-risk population, and are themselves particularly vulnerable to serious illness and death from COVID-19. Because the interventions are scalable, study results will interest a wide variety of audiences ranging from large public health institutions, LTC organizations, individual LTC facilities, and even to LTCWs themselves. We anticipate that the study outcomes will have the potential to change how vaccine confidence is addressed in LTC settings, not only for future COVID-19 vaccine adherence, but for other vaccination programs as well.

## Electronic supplementary material

Below is the link to the electronic supplementary material.

Additional file 1

Additional file 2

Additional file 3

Additional file 4

Additional file 5

Additional file 6

## Data Availability

Not applicable.

## List of abbreviations

***CBPR***: community-based participatory research
***CDC***: Centers for Disease Control and Prevention
***CFIR***: Consolidated Framework for Implementation Research
***CNA***: certified nursing assistant
***CONSORT***: Consolidated Standards of Reporting Trials
***COVID-19***: coronavirus disease 2019
***CPDE***: Center for Program Design and Evaluation
***CPHS***: Committee for the Protection of Human Subjects
***DSMB***: Data Safety and Monitoring Board
***ECU***: East Carolina University
***FRAME***: expanded framework for reporting adaptations and modifications to evidence-based interventions
***HTE***: heterogeneity of treatment effects
***IHI***: Institute for Healthcare Improvement
***LTC***: long-term care
***LTCW***: long-term care worker
***mRNA***: messenger ribonucleic acid
***NAHCA***: National Association of Health Care Assistants
***NPS***: net promoter score
***PCORI***: Patient Centered Outcomes Research Institute
***PI***: Principal Investigator
***RCT***: randomized controlled trial
***SAG***: Stakeholder Advisory Group
***SAGE***: Strategic Advisory Group of Experts
***SDM***: shared decision making
***SOP***: standard operating procedures
***SPIRIT***: Standard Protocol Items:Recommendations for Interventional Trials
***TSG***: Trial Steering Group
***VCI***: Vaccine Confidence Index
