# Supplementary material for: The CONFIDENT study protocol: a randomized controlled trial comparing two methods to increase long-term care worker confidence in the COVID-19 vaccines
Source: BMC Public Health. 2023 Feb 23;23:384. doi: 10.1186/s12889-023-15266-x (PMC9948785; doi:10.1186/s12889-023-15266-x)
Supplement: Supplementary file 2 — Additional file 2 [file 12889_2023_15266_MOESM2_ESM.pdf]

Additional File 2: Dialogue-Based Webinar Slides

CONFIDENT

Supporting long-term care workers during COVID-19

# LET'S TALK TOGETHER ABOUT COVID-19, VACCINES, AND BOOSTERS

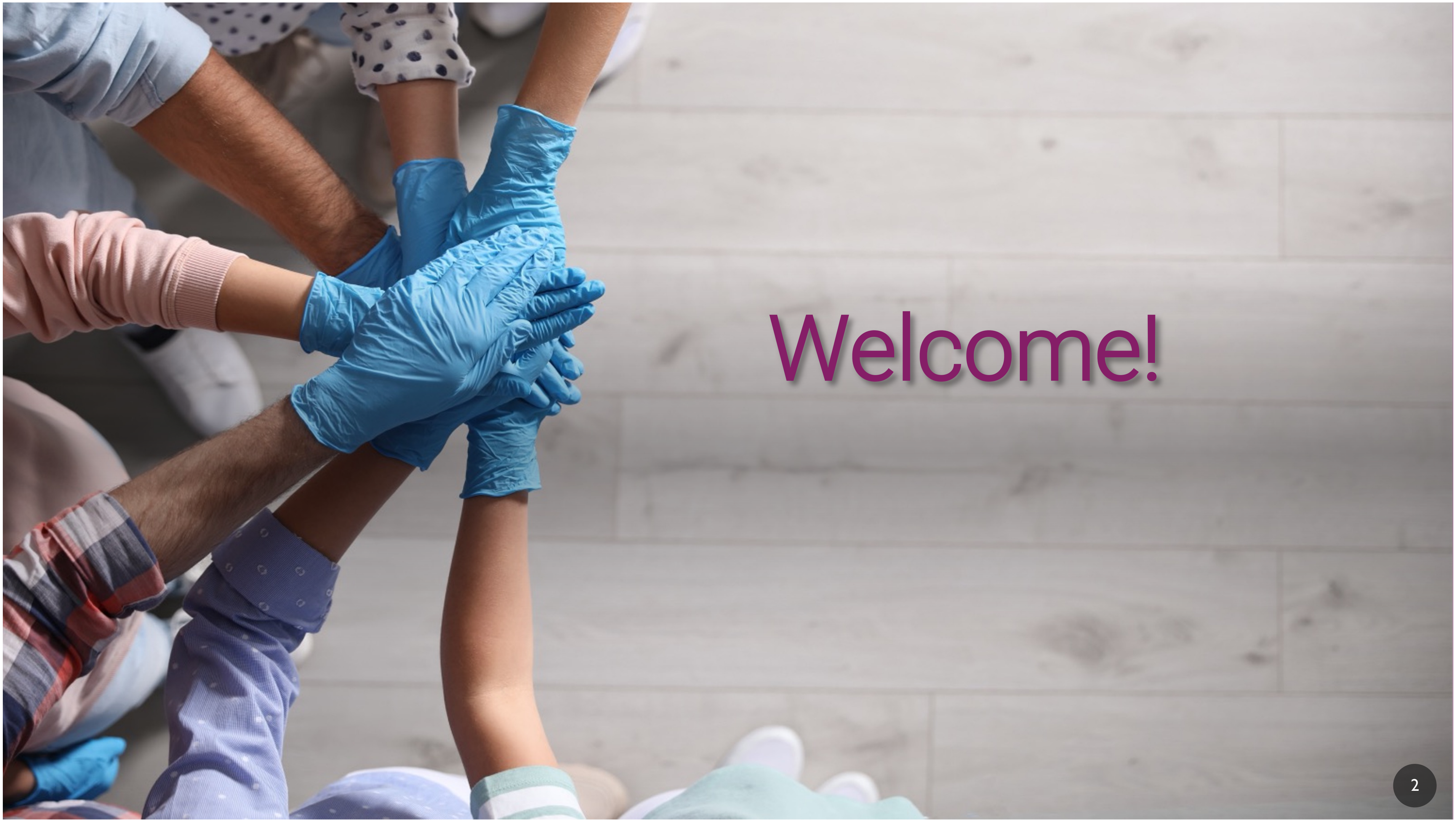

# Welcome!

# Using Zoom as a panelist

Please mute yourself unless you are talking

You can turn on your camera so we can see each other

Raise your Zoom hand or use the chat if you have a question or comment

Turn on live transcript if you want to read subtitles

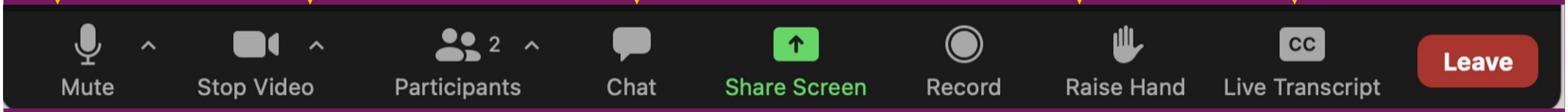


CONFIDENT
Supporting long-term care workers during COVID-19

# AGENDA

1. Welcome! (5 MIN)
2. Community standards.  (5 MIN)
3. A quick look at the Option Grid. (10 MIN)
4. Topic selection and discussion. (25 MIN)
5. Watch video. (2 MIN)
6. Closing remarks. (3 MIN)

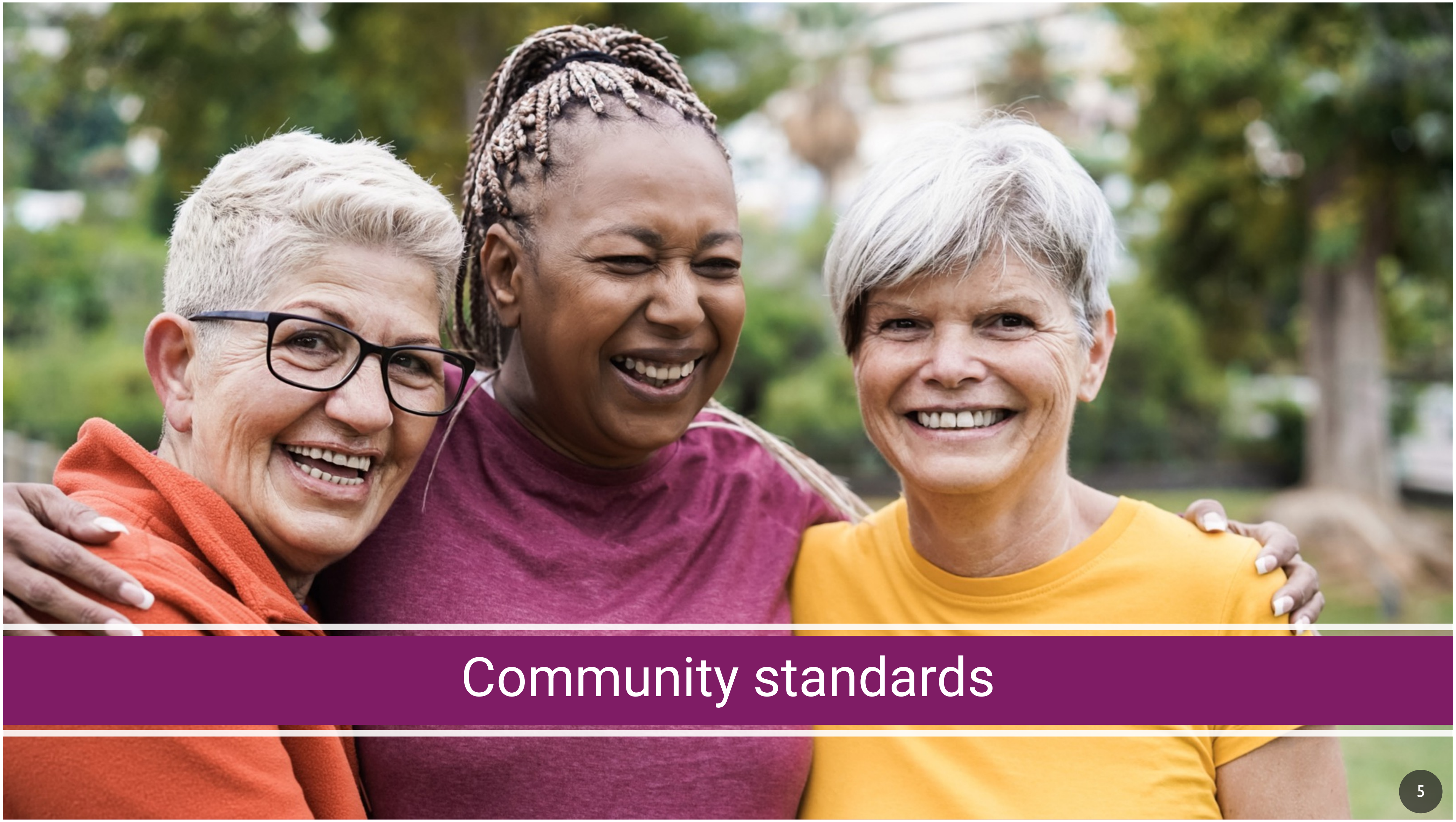
Community standards

# Community Standards

1. **No medical advice.** Please do not ask for or offer medical advice.
2. **No harassment.** It's ok to disagree, but please do not personally attack one another.
3. **No profanity or hate speech.** Please do not use profanity or racial/ethnic/religious/other slurs.
4. **No spam.** Please do not make too many off-topic comments. Avoid asking so many questions that others can't answer fast enough.
5. **No disclosure.** Please do not share any details about other webinar participants (names or any other personal information).

CONFIDENT
Supporting long-term care workers during COVID-19

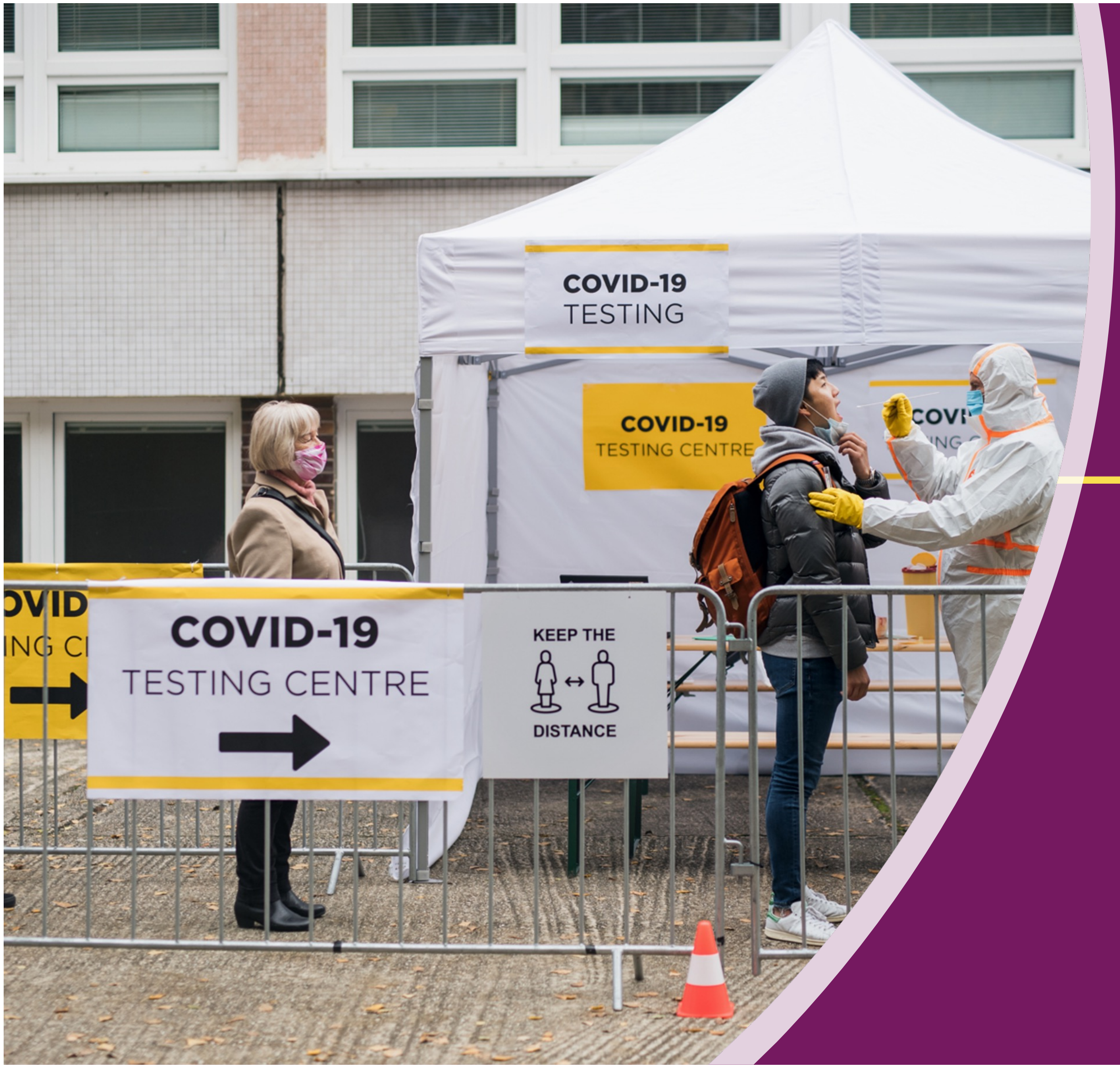


# A quick look at the Option Grid

optiongrid

| PATIENT QUESTIONS | No Vaccine | Pfizer/BioNTech and Moderna Vaccines | Johnson & Johnson/Janssen Vaccine | Novavax Vaccine |
|---|---|---|---|---|
| **What does the option involve?** | **Continue to distance**, avoid gatherings, wear a mask, and clean your hands often. | People **6 months and up** will get **2 shots in** their **arm.** For **Pfizer/BioNTech**, people **6 months to less than 5 years** will get **3 shots in** their **arm**. Each shot is given **3 to 8 weeks apart**. | People **18 years and up** will get **1 shot in their arm.** | People **12 years and up** will get **2 shots in their arm**, **3 to 8 weeks apart**. |
| **What are the benefits?** | You will **not have the side effects** that are common with the COVID-19 vaccine. | The vaccine helps lower the risk of getting COVID-19. It also **helps protect** people **from serious illness** if they get COVID-19.<br>**2 weeks** after having all your **shots**, it is **safer to do things you did before the pandemic.** You should **still follow guidance** about wearing a mask and distancing.<br>Being fully vaccinated **helps keep COVID-19 under control**. | The vaccine helps lower the risk of getting COVID-19. It also **helps protect** people **from serious illness** if they get COVID-19.<br>**2 weeks** after the **shot**, it is **safer to do things you did before the pandemic.** You should **still follow guidance** about wearing a mask and distancing.<br>Being fully vaccinated **helps keep COVID-19 under control**. | The vaccine helps lower the risk of getting COVID-19. It also **helps protect** people **from serious illness** if they get COVID-19.<br>**2 weeks** after the **second shot**, it is **safer to do things you did before the pandemic.** You should **still follow guidance** about wearing a mask and distancing.<br>Being fully vaccinated **helps keep COVID-19 under control**. |

| PATIENT QUESTIONS | No Vaccine | Pfizer/BioNTech and Moderna Vaccines | Johnson & Johnson/Janssen Vaccine | Novavax Vaccine |
|---|---|---|---|---|
| **What are the short-term side effects or harms?** | You will be at **higher risk of getting COVID-19**.<br>**Symptoms** of COVID-19 include:<br>• feeling tired.<br>• fever or chills.<br>• body aches.<br>• shortness of breath and cough.<br>• problems with taste or smell.<br>COVID-19 can also lead to **serious illness**. This can result in a **hospital stay, needing a machine to breathe**, or **death**. | **Common side effects** within the first 3 days include:<br>• feeling tired.<br>• fever or chills.<br>• body aches.<br>• headache.<br>• soreness from the shot.<br>These effects are **more likely with the second shot** and are normal effects the vaccine can have. They typically **go away in 1 to 2 days**.<br>**Serious side effects or harms are rare**. | **Common side effects** within the first 3 days include:<br>• feeling tired.<br>• fever.<br>• body aches.<br>• headache.<br>• soreness from the shot.<br>These are the normal effects the vaccine can have and typically **go away in 1 to 2 days**.<br>**Serious side effects or harms are rare**. | **Common side effects** within the first 3 days include:<br>• feeling tired.<br>• fever.<br>• body aches.<br>• headache.<br>• soreness from the shot.<br>These effects are **more likely with the second shot** and are normal effects the vaccine can have. They typically **go away in 1 to 2 days**.<br>**Serious side effects or harms are rare.** |
| **What are the long-term side effects or harms?** | Sometimes short-term **symptoms from COVID-19 can last for a long time**. Some people get new symptoms that can include hair loss, trouble with focus or memory, or lung damage. | There are no long-term studies on COVID-19 vaccines. But **side effects or harms from vaccines are uncommon after 6 weeks.** | There are no long-term studies on COVID-19 vaccines. But **side effects or harms from vaccines are uncommon after 6 weeks.** | There are no long-term studies on COVID-19 vaccines. But **side effects or harms from vaccines are uncommon after 6 weeks.** |

| PATIENT QUESTIONS | No Vaccine | Pfizer/BioNTech and Moderna Vaccines | Johnson & Johnson/Janssen Vaccine | Novavax Vaccine |
|---|---|---|---|---|
| **Should I get a booster?** | Does not apply | The CDC recommends an **updated (bivalent) booster at least 2 months after** the second dose or the original (monovalent) booster, for **people 5 years and up**. | The CDC recommends an **updated (bivalent) booster at least 2 months after** the first dose or the original (monovalent) booster.<br>Only the **Pfizer/BioNTech and Moderna** vaccines are **approved** for boosters. | The CDC recommends an **updated (bivalent) booster at least 2 months after** the first dose or the original (monovalent) booster.<br>Only the **Pfizer/BioNTech and Moderna** vaccines are **approved** for boosters. |
| **What else do I need to know?** | If you do not want a vaccine now but change your mind later, you can get it then. | • The vaccine **cannot give you COVID-19**.<br>• The vaccine **cannot change your DNA**.<br>• There is **no microchip** in the vaccine.<br>• If you have had COVID-19, the **vaccine may still lower your risk** of getting it again. | • The vaccine **cannot give you COVID-19**.<br>• The vaccine **cannot change your DNA**.<br>• There is **no microchip** in the vaccine.<br>• If you have had COVID-19, the **vaccine may still lower your risk** of getting it again. | • The vaccine **cannot give you COVID-19**.<br>• The vaccine **cannot change your DNA**.<br>• There is **no microchip** in the vaccine.<br>• If you have had COVID-19, the **vaccine may still lower your risk** of getting it again. |

## Any questions about the Option Grid?

CONFIDENT
Supporting long-term care workers during COVID-19

https://decisions.dynamed.com/shared-decision-making/covid-19-vaccine-options

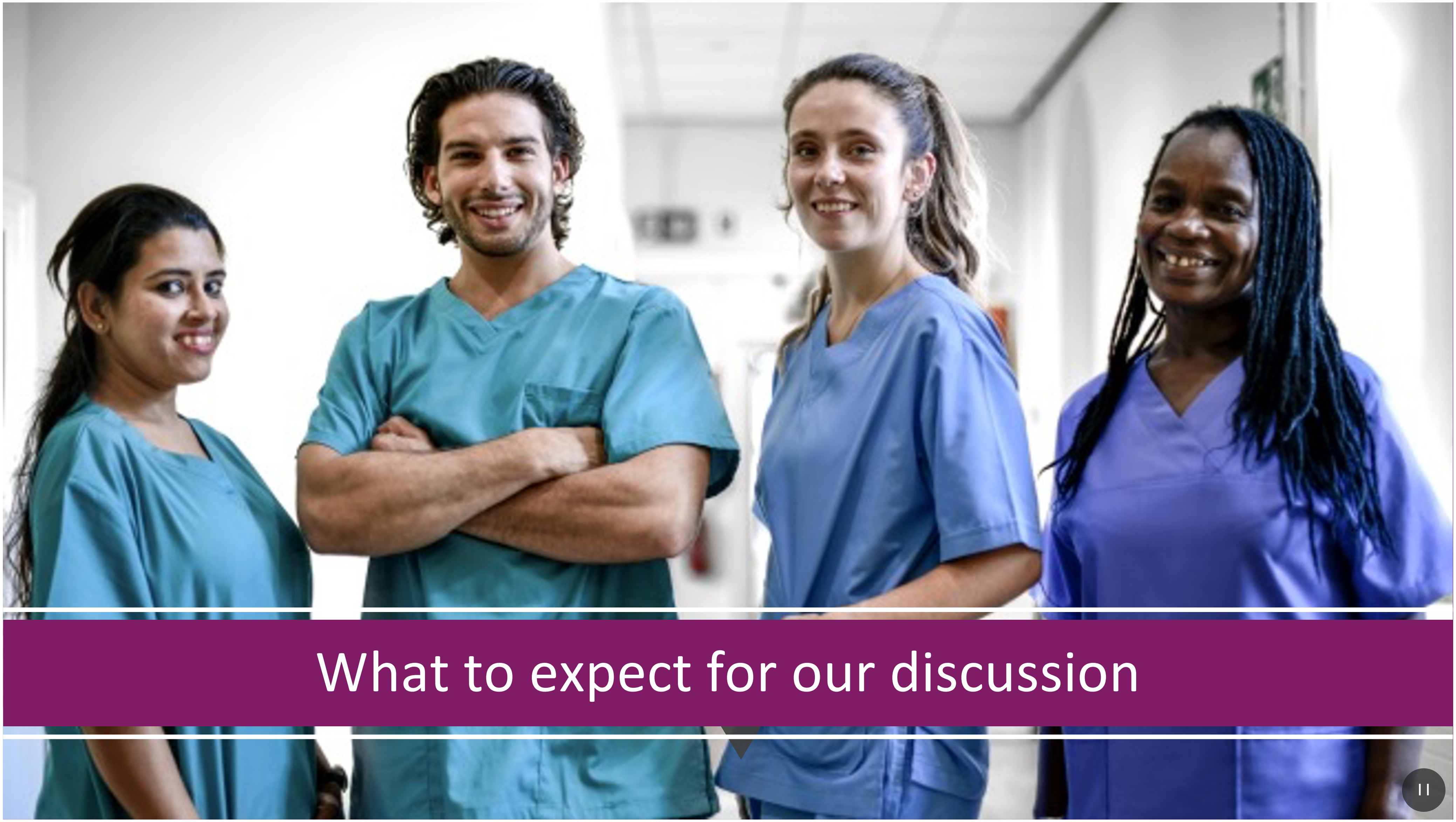

# What to expect for our discussion

# Discussion guidelines

- We are in this together and want to address your concerns.
- We value everyone's perspectives and expertise.
- We allow everyone the chance to speak and avoid dominating the discussion.
- We share personal experiences rather than stereotypes.
- We listen and avoid making judgmental comments.
- Don't interrupt others (raise Zoom hand or use chat instead).
- The chat is an extension of our discussion.

# Topic selection

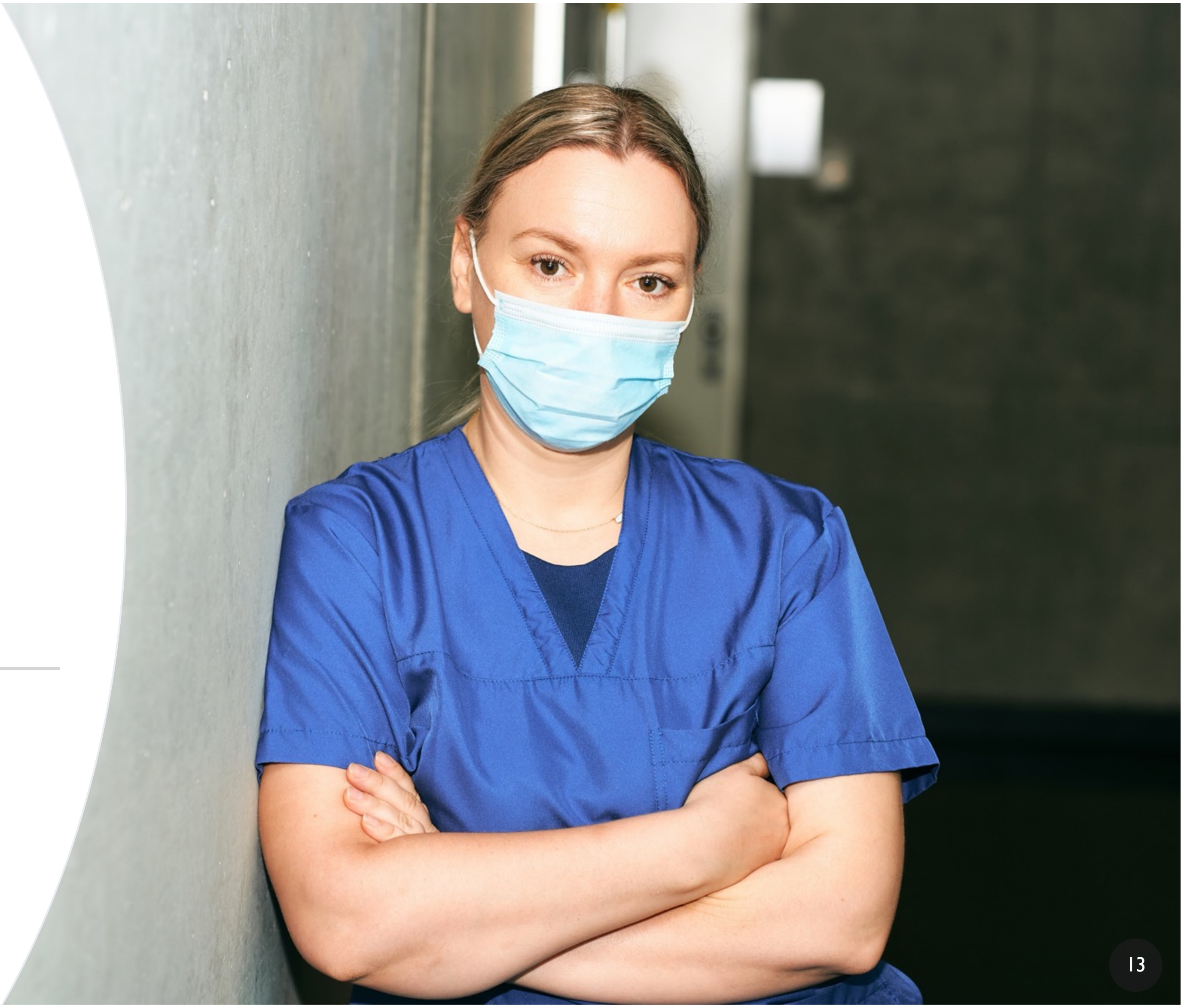

# Discussion topic options

| Vaccine and booster creation | Vaccine and booster side effects and risks | Vaccine and booster benefits | COVID-19 in general |
|---|---|---|---|
| (FDA approval, speed of development) | (Short and long term risks) | (Lowering infection rates, preventing illness and death) | (COVID-19 deaths, variants, where are we at) |

CONFIDENT
Supporting long-term care workers during COVID-19

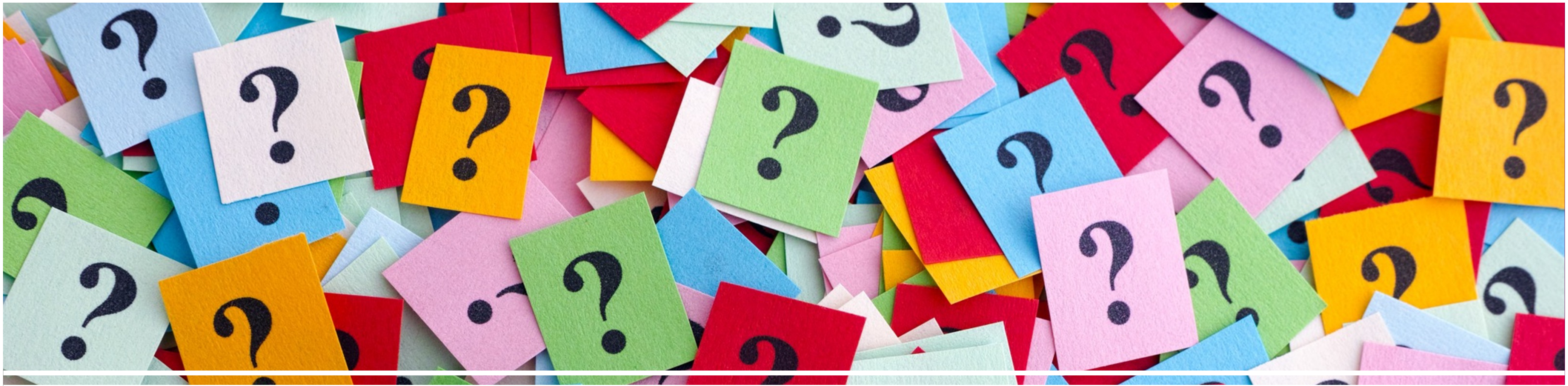

# Discussion

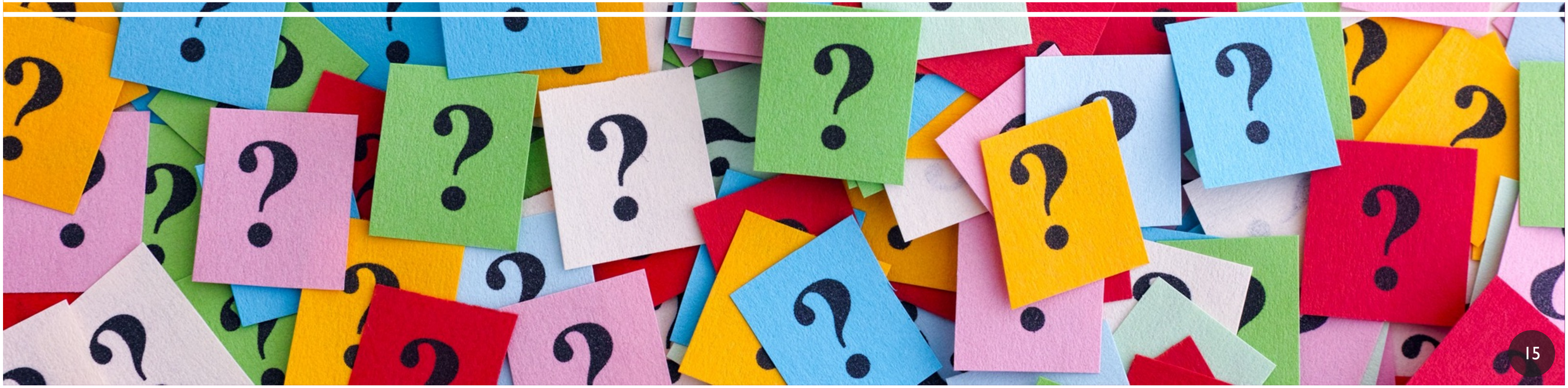

[Resident video plays here]

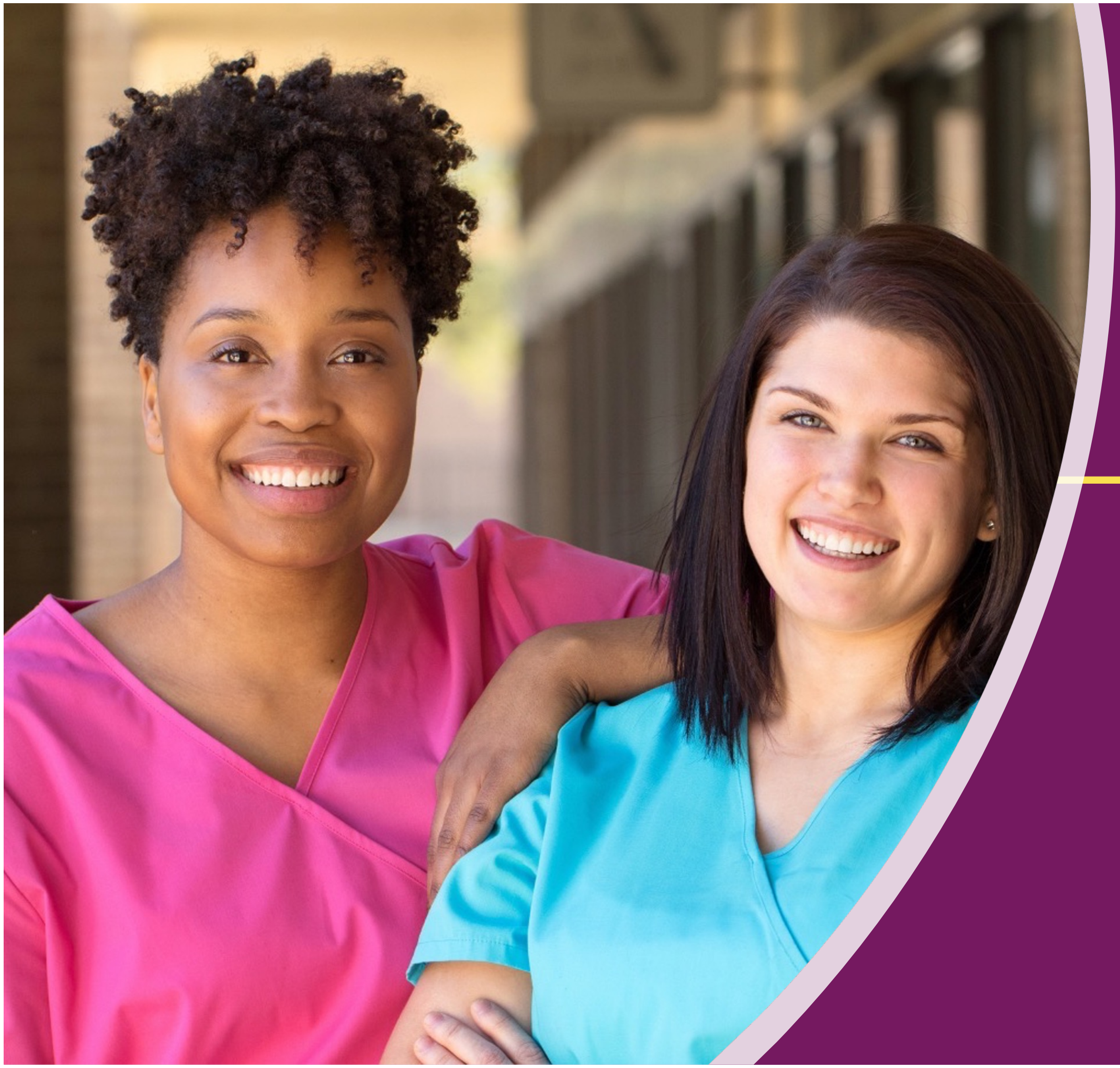

# Thank you!

If you have questions or concerns about this discussion session or the study, please contact confident.study@dartmouth.edu
