# Supplementary figures and images for: The CONFIDENT study protocol: a randomized controlled trial comparing two methods to increase long-term care worker confidence in the COVID-19 vaccines
Source: BMC Public Health. 2023 Feb 23;23:384. doi: 10.1186/s12889-023-15266-x (PMC9948785; doi:10.1186/s12889-023-15266-x)

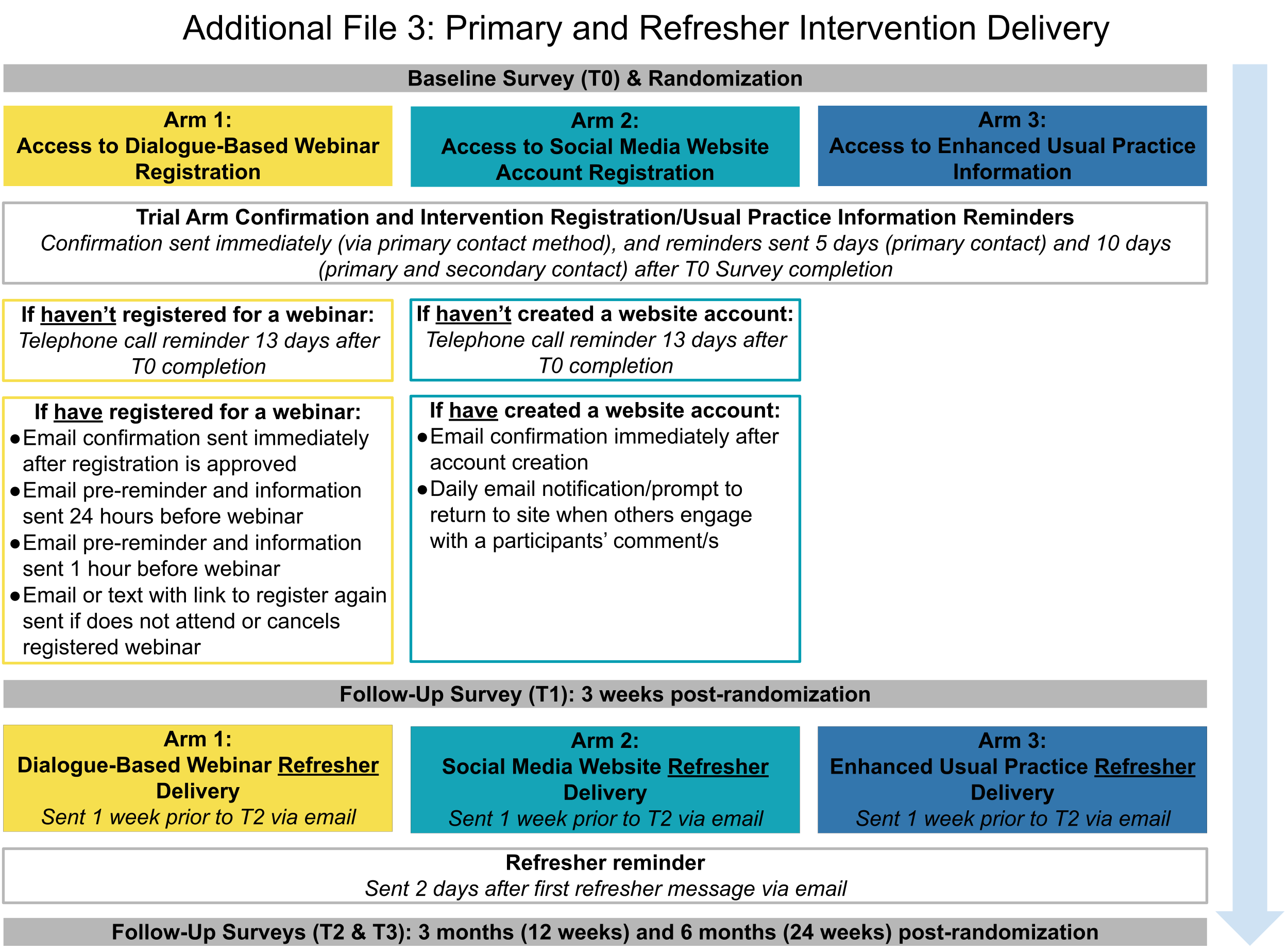

Supplement: Supplementary file 3 — Additional file 3 [file 12889_2023_15266_MOESM3_ESM.png]
