# Supplementary material for: The CONFIDENT study protocol: a randomized controlled trial comparing two methods to increase long-term care worker confidence in the COVID-19 vaccines
Source: BMC Public Health. 2023 Feb 23;23:384. doi: 10.1186/s12889-023-15266-x (PMC9948785; doi:10.1186/s12889-023-15266-x)
Supplement: Supplementary file 4 — Additional file 4 [file 12889_2023_15266_MOESM4_ESM.pdf]

Additional File 4: Data Analysis Methodology
Study Aims 1 and 2

To be read in conjunction with the study protocol manuscript: “The CONFIDENT study protocol: a randomized controlled trial comparing two methods to increase long-term care worker confidence in the COVID-19 vaccines”

Data collection summary

Below is a summary table of primary and secondary outcomes and other data collected (including demographic characteristics, contextual factors, and intervention engagement) and corresponding time points for analyses.

Table 1. Time points for outcome and other data assessment

**Data Category**	**Name of Outcome / Data**	**Time Point / Survey**			
		**T0 baseline**	**T1 3 weeks post-baseline**	**T2 3 months post-baseline**	**T3 6 months post-baseline**
Participant characteristics	Age, gender, zip code, race/ethnicity, education, insurance status, health literacy, religiosity, extent influenced by others, long-term care (LTC) role, duration of experience in LTC, baseline vaccination status	✓			
Contextual factors	Personal experiences related to COVID-19, COVID-19 vaccines, and other vaccines	✓	✓	✓	✓
Intervention and usual practice engagement*	Engagement with each intervention and enhanced usual practice information (including refreshers)		✓	✓	✓
Primary outcome	COVID-19 vaccine confidence		✓		

Secondary outcomes	Change from baseline in COVID-19 vaccine confidence	✓	✓	✓	✓
	COVID-19 vaccine uptake (any dose)		✓		
	COVID-19 vaccine uptake (initial series completion)		✓		
	COVID-19 vaccine uptake (booster completion)		✓		
	Likelihood of recommending (promoting) COVID-19 vaccination		✓		
	Likelihood of recommending (promoting) COVID-19 booster vaccination to coworker		✓		
	COVID-19 vaccine intent (initial series)		✓		
	COVID-19 vaccine intent (booster)		✓		
	COVID-19 vaccine intent (future vaccine recommendations)		✓		
	Feeling informed about the COVID-19 vaccines		✓		
	Identification of COVID-19 vaccine information and misinformation		✓		
	Trust in COVID-19 vaccine information from different sources		✓		
	Change from baseline in secondary outcomes	✓	✓	✓	✓
	As treated analyses of primary and secondary outcomes	✓	✓	✓	✓

*Data on intervention engagement will be collected via a combination of online activity data and participant survey questions

Statistical principles

Confidence intervals and p-values

P-values ≥0.0001 will be reported to 4 decimal places; p-values less than 0.0001 will be reported as “<0.0001”. The mean, standard deviation, and any other statistics other than quantiles, will be reported to one decimal place greater than the original data. Quantiles, such as median, minimum, or maximum will use the same number of decimal places as the original data. Estimated parameters, not on the same scale as raw observations (e.g. regression coefficients) will be reported to 3 significant figures.

Adherence to interventions

Analysis of both intention-to-treat (ITT) and as-treated populations will be performed. All participants who are randomized to a trial arm will be included in the ITT analysis. Participants who adhere to major engagement criteria for their primary intervention and/or refresher will be included in the as-treated analyses. We will utilize both online activity and participant self-reported survey data (see manuscript for details on data collected) to develop criteria for determining whether each participant was ‘engaged’ or ‘not engaged’ with their respective primary and refresher intervention. As-treated analyses will be performed according to the following prespecified groups:

1. Engaged with primary intervention + engaged with refresher
2. Engaged with primary intervention + did not engage with refresher
3. Did not engage with primary intervention + engaged with refresher
4. Did not engage with primary intervention + did not engage with refresher

Analysis methodology

Aim 1

To compare the impact of two interventions delivered online: 1) a dialogue-based webinar (webinar) using the existing COVID-19 Option Grid conversation aid and, 2) an interactive and, dynamic, and multi-component social media web application (social media website), compared to enhanced usual practice (link to Centers for Disease Control (CDC) vaccine website), on COVID-19 vaccine confidence (primary outcome), and other secondary outcomes among LTCWs. We hypothesize that each intervention will be superior to enhanced usual practice at increasing vaccine confidence (*Hypothesis 1.1*) and that the dialogue-based webinar intervention will be superior to the social media arm at increasing vaccine confidence (*Hypothesis 1.2*).

# Corresponding analyses

Primary outcome

The primary outcome is the binary classifier of vaccine confidence derived from the three-item Vaccine Confidence Index (VCI), evaluated at 3 weeks post-randomization, which will be analyzed by randomization arm. The determined binary predictors of intervention outcomes will be compared using two-proportion z-tests to identify significant differences between each intervention and enhanced usual practice (Arm 1 vs Arm 3 & Arm 2 vs Arm 3 - superiority analyses) and between the intervention arms (Arm 1 vs Arm 2 - two-tailed equivalency analysis).

Secondary outcomes

- *Change from baseline in COVID-19 Vaccine Confidence.* Analysis of change from baseline will be based on a binary classifier whether the average score increases (1) or does not increase (2) at T1, T2, and T3 versus baseline.

- *Likelihood of recommending COVID-19 vaccination (others not vaccinated).* This question will be evaluated using a Net Promoter Score (NPS) approach, adding one for each positive response, zero for neutral, and subtracting one for each negative response, then dividing by the number of non-missing responses for each trial arm. NPS values will be compared using Wald intervals [1].

- *Likelihood of recommending COVID-19 booster vaccination (coworker).* This question will be evaluated using the NPS approach, described above.

- *COVID-19 vaccine uptake (any dose).* This will be evaluated as a binary classifier compared across trial arms using two-proportion z-tests.

- *COVID-19 vaccine uptake (initial series completion).* For participants who have received any dose of a COVID-19 vaccine, this will be evaluated as a binary classifier compared across trial arms using two-proportion z-tests.

- *COVID-19 vaccine uptake (booster completion).* For participants who have completed their initial vaccine series, this will be evaluated as a three category variable (No, Not sure, Yes) compared across trial arms using chi-squared tests.

- *COVID-19 vaccine intent (initial series)*. For participants who are unvaccinated, we will assess their intentions of getting a COVID-19 vaccine, this will be evaluated as a three category variable (No, Not sure, Yes) compared across trial arms using chi-squared tests.

- *COVID-19 vaccine intent (booster)*. For participants who have completed an initial vaccine series, this will be evaluated as a three category variable (No, Not sure, Yes)

compared across trial arms using chi-squared tests.

- *COVID-19 vaccine intent (future vaccine recommendations).* Participants' intent to get COVID-19 vaccines regularly in the future if they are recommended will be evaluated as a three category variable (No, Not sure, Yes) compared across trial arms using chi-squared tests.

- *Feeling informed about the COVID-19 vaccines.* The degree to which participants' feel informed about the COVID-19 vaccines (have enough information and understand that information) will be evaluated using a continuous variable, calculated as the mean scale score across two items [(1) having enough information, (2) understanding the information] for each participant. Results will be compared across trial arms with two-sample unpaired t-tests.

- *Identification of COVID-19 vaccine information and misinformation.* Subjects' identification of COVID-19 vaccine information and misinformation will be scored on a scale of zero to four and treated as a continuous linear variable. Results will be compared across trial arms with two sample unpaired t-tests.

- *Trust in COVID-19 information from different sources.* Subjects' trust in COVID-19 information from different sources will be evaluated using a continuous variable, calculated as the mean scale score across three items for each participant. Results will be compared across trial arms with two-sample unpaired t-tests.

- *Change from baseline in secondary outcomes.* Change from baseline will be scored as a binary variable with an increased value scored as one and a no change or decreased value scored as zero. For each evaluation after baseline, each arm will be compared using two-proportion z-tests.

- *As-treated analysis of primary and secondary outcomes.* Primary and secondary outcome analyses will be repeated, limited to the as treated samples.

Aim 2

To determine if LTCWs' characteristics and other factors mediate and moderate the interventions' impact on vaccine confidence and other secondary outcomes.

We hypothesize that increased perceptions of feeling informed about the vaccines, identification of vaccine information and misinformation, and trust in vaccine information provided by different sources will explain (mediate) the relationship between the interventions and vaccine confidence, as well as other secondary outcomes (*Hypothesis 2.1)*.

We will also conduct exploratory heterogeneity of treatment effects (HTE) analyses to identify whether certain participant characteristics and beliefs moderate the relationships among each of

the interventions and vaccine confidence, as well as other secondary outcomes. Variables to be explored will include, but are not limited to, baseline vaccination status, religious beliefs, perceived influence of others, age, race, ethnicity and personal experiences with COVID-19.

Corresponding analyses

Mediation analysis

We hypothesize that the relationship between participants' assigned trial arm and primary and secondary outcomes will be mediated by the effects of feeling informed, trust in vaccine information, and beliefs about vaccines (measured through questions to determine each participant's ability to correctly classify vaccine information and misinformation). Responses for these three variables will be evaluated at each time point versus outcomes by randomization arm to identify differences by arm using the Kruskal-Wallis test. Significant relationships will be explored for mediation effects in the primary and secondary outcome analyses using the method of Barron and Kenny [2]. If this results in a significant improvement in model prediction, hypothesis 2.1 will be supported.

Heterogeneity of treatment effects/Moderation analysis

To understand how the relationship between study interventions and outcomes are moderated by participant characteristics including age, gender, location (level of regional analysis selected for appropriate granularity), educational attainment, race and ethnicity, health insurance status, health literacy, religiosity, LTCW role, duration of experience in long-term care, extent influenced by others regarding COVID-19 vaccination, and baseline vaccination status, we will evaluate the primary and secondary outcomes using linear and logistic regression in a mixed-effects model.

Exploratory analyses

Change from baseline in COVID-19 Vaccine Confidence. Looking deeper at the change in vaccine confidence, analyses will consider (a) the proportion of positive (agree and strongly agree) responses in the VCI, (b) positive responses in each of the three VCI questions. Significant correlates with demographic, contextual, and study variables will be evaluated.

External contextual factors. Outside of the study surveys and throughout the trial, we will monitor external factors that may impact participants' views and actions towards the COVID-19 vaccines. This may include monitoring policy and mandate changes for LTCWs and changes in the nature of the pandemic, among other things.

Additional analyses. Any unvalidated thresholds utilized in the primary or secondary analyses will be evaluated for sensitivity to the choice of threshold in ROC analyses. Nonlinear higher-dimensional effects and interactions among variables will be identified by identifying positive or negative outcomes grouped around specific baseline characteristics that are highly unlikely to result from random chance.

Duplicate and missing data

Participants must complete a baseline (T0) survey to be considered enrolled in the study. In the event of duplicate complete T0 submissions, the first complete survey will be used. For participants who complete duplicate follow-up surveys (T1, T2 or T3), the first complete survey will also be used.

The study team will endeavor to minimize missing data; however, it is possible that efforts to ensure complete data collection within the response windows will be unsuccessful or because participants may choose not to respond to one or more questions. Should any primary and/or secondary outcome variables have >5% missing data, the reasons for any missing data will be considered through an analysis for common baseline characteristics of individuals with specific responses missing, those who were lost to follow-up, and those who did not respond to one or more evaluations but were not lost to follow-up. Specific questions with significantly higher rates of missing data will be reviewed for wording bias or missing response options. Based on these considerations, a stochastic outcome set model will be created using multiple imputation and Bayesian methods, as appropriate, to provide a high confidence range for imputed values. Using this approach, an expected result and confidence intervals will be produced for the missing data and the resulting analysis of the full data set, taking into account the statistical uncertainty attributed to the missing data. If available, data from both baseline and subsequent follow-up evaluations will be used in imputing missing results (e.g., responses from T0 and T2 evaluations could be used to impute T1 responses) through multiple imputation regression modeling [3].

Response rates and the frequency of missing data in the ITT and as-treated populations will be reported with the results.

Fraudulent enrollment

Identity verification of some participants will occur after they have completed their journey in the study (see manuscript 'Procedures and data collection, Verification process'). We will also conduct a rigorous review of survey metadata (e.g., IP addresses) and other responses to identify duplicate survey completions or other suspicious survey activity [4,5]. Upon discovery of compelling evidence of fraudulent or illegitimate enrollment, such subjects will be excluded from the study prior to any outcome analyses.

Methods to minimize bias

A naïve analysis will be conducted at the end of the study based only on the unmodified data reflecting ITT assignments of eligible subjects to establish an initial estimator. All primary and secondary analyses will be conducted based on the statistical analysis plan.

Multiple testing

The primary outcome involves three separate tests, one a two-tailed equivalence test and two one-tailed superiority tests. In order to achieve a family-wise error rate less than 0.050, the Bonferonni method was employed to set a p-value threshold at 0.016 for each of the three primary outcome analyses. For each comparison, mean-value separation will be considered significant based on a 98.4% confidence interval, which translates to +/- 2.45 times the standard error for the sample mean for the equivalence test and greater than 2.15 times the standard error for the superiority analyses.

References

1. B. Rocks, Interval Estimation for the “Net Promoter Score.” *Am. Stat.* 70, 365–372 (2016).
2. R. M. Baron, D. A. Kenny, The moderator–mediator variable distinction in social psychological research: Conceptual, strategic, and statistical considerations. *J. Pers. Soc. Psychol.* **51**, 1173–1182 (1986).
3. T.E. Raghunathan, J.M. Lepkowski, J. Van Hoewyk, P. Solenberger, et al. A multivariate technique for multiply imputing missing values using a sequence of regression models. *Surv. Methodol.* 27, 85-96 (2001).
4. Stevens G, Washburn H, Theiler R, Woodhams E, Donnelly K, Thompson R. Enrolment fraud in online shared decision-making research: Lessons learned from an internet-based randomised controlled trial. Oral presentation at the *10th International Shared Decision-Making Conference (ISDM)*, Quebec City, Canada. https://fourwaves-sots.s3.amazonaws.com/static/media/uploads/2019/06/28/isdm2019-oralsessionsbooklet-2019-06-28.pdf. Accessed 17 Dec 2022.
5. Teitcher JEF, Bockting WO, Bauermeister JA, Hoefer CJ, Miner MH, Klitzman RL. Detecting, Preventing, and Responding to “Fraudsters” in Internet Research: Ethics and Tradeoffs. *J Law Med Ethics*. 2015;43:116–33.
