# Supplementary material for: The CONFIDENT study protocol: a randomized controlled trial comparing two methods to increase long-term care worker confidence in the COVID-19 vaccines
Source: BMC Public Health. 2023 Feb 23;23:384. doi: 10.1186/s12889-023-15266-x (PMC9948785; doi:10.1186/s12889-023-15266-x)
Supplement: Supplementary file 5 — Additional file 5 [file 12889_2023_15266_MOESM5_ESM.pdf]

Additional File 5: Aim 3 Planned Activities

To be read in conjunction with the study protocol manuscript: “The CONFIDENT study protocol: a randomized controlled trial comparing two methods to increase long-term care worker confidence in the COVID-19 vaccines”

Step	When	Activity	Objectives	Participants	Methods
Task 1. Conduct an intervention and implementation needs assessment					
**Step 1**	Study set-up	Adopter, Implementer, Maintainer [1] brainstorm	Determine who will participate in the implementation consultations	Study team, including those at collaborating organizations	Group meetings and consultations (convenience sampling)
**Step 2**	Study set-up	Intervention consultations<br><br>*1st round*	Assist in the development of the two study interventions. Includes asking about their own information needs, views and experiences with COVID-19/vaccines, and providing feedback on proposed intervention content and functionalities, among other things.	Long-term care workers (LTCWs) on our Stakeholder Advisory Group (SAG)	Interviews (one-on-one, video-based) (convenience sampling)
				Adults from the general public (demographically representative of LTCWs) recruited by Qualtrics Panel Services	Online survey (purposive sampling)
Task 2A. Finalize intervention content and delivery using co-design/user-centered design principles					
**Step 3**	Study set-up	Intervention consultations<br><br>*2nd round (as needed)*	Continuation of Step 2, to further refine and finalize the interventions. Informal feedback on intervention design and user-testing of interventions in iterative cycles.	LTCWs on our SAG, other LTCWs not associated with the study, and study team members/collaborators	Emails, study meetings, polls, user-testing sessions (convenience sampling)

Task 2B. Identify adoption and implementation outcomes, performance objectives, determinants, and change objectives					
**Step 4**	Study set-up	Implementation consultations<br><br>*1st round*	Determine what is required to effectively implement the two interventions during the trial, and how implementation success should be measured. Also explore how we might sustain the interventions once the trial is completed.	Study team, including those at collaborating organizations, consultants, and SAG members	Interviews (likely multiple rounds, one-on-one, video-based or telephone) (convenience sampling)
Task 3. Select theoretical methods and design implementation strategies					
**Step 5**	Study set-up	Implementation collaboration	Identify necessary actions to facilitate implementation and sustainability using the Consolidated Framework for Implementation Research (CFIR) [2, 3].	Study team, including those at collaborating organizations, consultants, and SAG members	Group meetings and consultations (convenience sampling)
Task 4. Produce implementation protocols and materials					
**Step 6**	Study set-up	Implementation collaboration	Using the information gleaned from Tasks 1-3, develop implementation protocols and materials.	Study team, including those at collaborating organizations, consultants, and SAG members	Group meetings and consultations (convenience sampling)
Task 5. Select assessment metrics					
**Step 7**	Study set-up and trial phase	Implementation collaboration	Collaboratively generate process evaluation questions and design evaluation assessment processes for Aim 3 interviews during and post-trial.	Study team, including those at collaborating organizations, consultants, and SAG members	Group meetings and consultations (convenience sampling)
**Step 8**	Trial phase	Implementation consultations<br><br>*2nd round*	Determine views on the interventions, involvement in co-designing the implementation process (if relevant), and views on adaptations and the future sustainability of the interventions.	Trial participants, study team members, and other key stakeholders identified in Step 1	Interviews (convenience and purposive sampling)

**Step 9**	Trial and sustainability phases	Implementation assessment	Determine the success of the intervention implementation.	May include study team members, trial participants, and others TBD	Interviews, surveys (convenience and purposive sampling), online activity data, field notes

References

1. Fernandez ME, Ten Hoor GA, van Lieshout S, Rodriguez SA, Beidas RS, Parcel G, et al. Implementation Mapping: Using Intervention Mapping to Develop Implementation Strategies. Front Public Health. 2019;7:158.

2. Kirk MA, Kelley C, Yankey N, Birken SA, Abadie B, Damschroder L. A systematic review of the use of the Consolidated Framework for Implementation Research. Implement Sci. 2016;11:1–13.

3. Damschroder LJ, Aron DC, Keith RE, Kirsh SR, Alexander JA, Lowery JC. Fostering implementation of health services research findings into practice: a consolidated framework for advancing implementation science. Implement Sci. 2009;4:50.
