# Supplementary material for: The CONFIDENT study protocol: a randomized controlled trial comparing two methods to increase long-term care worker confidence in the COVID-19 vaccines
Source: BMC Public Health. 2023 Feb 23;23:384. doi: 10.1186/s12889-023-15266-x (PMC9948785; doi:10.1186/s12889-023-15266-x)
Supplement: Supplementary file 6 — Additional file 6 [file 12889_2023_15266_MOESM6_ESM.pdf]

Additional File 6: Progress of Research

To be read in conjunction with the study protocol manuscript: “The CONFIDENT study protocol: a randomized controlled trial comparing two methods to increase long-term care worker confidence in the COVID-19 vaccines”

The diagram below provides a timeline of events that required adaptations to our study protocol and recruitment and verification processes.

Protocol Adaptation Timeline (2022)

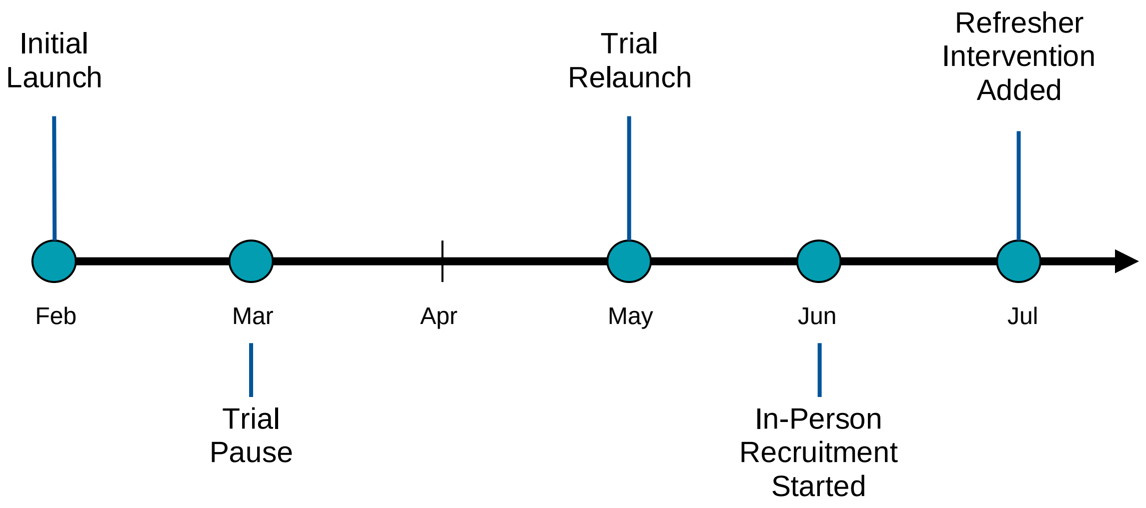

Initial Launch and Trial Pause. We initially launched the trial and began recruitment in February 2022. After 3 weeks, we encountered significant suspected enrollment fraud, necessitating a pause in study recruitment and enrollment. Assisted by prior guidance for detecting and responding to enrollment fraud [1,2] and in consultation with our IRB and DSMB, we addressed the situation and removed those who we could not confirm were legitimate long-term care workers (LTCWs). Prior to pausing the trial, we also had encountered high screen-out rates on the eligibility criterion assessing vaccine worry, which we attributed to the changing COVID-19 context, increasing vaccination rates, and federal mandates.

As a result of these concomitant challenges, we 1) developed a more rigorous verification process (see ‘Procedures and data collection, Verification process’ in the protocol manuscript), and 2) adapted our eligibility criteria to allow those who had not received a COVID-19 booster to enroll, regardless of their level of vaccine worry (see ‘Participants’ in the protocol manuscript).

Trial Relaunch. We relaunched the trial in May 2022. We plan to treat all data collected in the initial verified recruitment sample (n=25) as pilot data (to be excluded from study analyses),

given the differences in screening criteria, verification processes, and elapsed time between the two groups.

In-Person Recruitment Started. Guided by our stakeholder partners and COVID-19 restrictions at the time of our study start, we had planned a rigorous online recruitment strategy through email invitations and social media posts. However, due to lower than expected activity from these recruitment channels, we moved to social media paid advertising (Facebook and Instagram). Contending with the fraudulent enrollment issue aside, this has and will enable us to reach a wider audience. However, unanticipated issues targeting non-CNAs via these channels, coupled with the lessening of COVID-19 restrictions, provided the impetus for adding in-person recruitment in June 2022 to increase participant diversity.

Refresher Intervention Added. We encountered another study complication in June 2022, when low engagement with the webinar intervention surfaced. Several options and the pros and cons of each were judiciously considered, with the aims of 1) exposing more LTCWs to information they may have missed, and 2) being generalizable and transferable with the potential for scale up and implementation. Adding a refresher intervention across all trial arms offered a feasible and pragmatic way to increase engagement without introducing bias across one or more trial arms. In July 2022, we began emailing refresher content to participants one week prior to their receipt of the second follow-up survey (T2).

Continued Fraudulent Activity. Despite developing a rigorous process to verify a participant's status as a LTCW, we continued to experience more sophisticated and nuanced fraudulent activity, including cases of impersonation. As a result, in mid-October 2022, we added identity checks to our verification process (in addition to our LTCW verification), by implementing Trans Union's TLOxp, a verification service. The use of TLOxp and our process for participant identity verification was informed by prior work [3]. We also plan to retrospectively verify the identity of participants enrolled in the study prior to mid-October 2022 (before our implementation of TLOxp) to ensure integrity of study data.

References

1. Stevens G, Washburn H, Theiler R, Woodhams E, Donnelly K, Thompson R. Enrolment fraud in online shared decision-making research: Lessons learned from an internet-based randomised controlled trial. Oral presentation at the *10th International Shared Decision-Making Conference (ISDM)*, Quebec City, Canada. https://fourwaves-sots.s3.amazonaws.com/static/media/uploads/2019/06/28/isdm2019-oralsessionsbooklet-2019-06-28.pdf. Accessed 17 Dec 17 2022.
2. Teitcher JEF, Bockting WO, Bauermeister JA, Hoefer CJ, Miner MH, Klitzman RL. Detecting, Preventing, and Responding to “Fraudsters” in Internet Research: Ethics and Tradeoffs. J Law Med Ethics. 2015;43:116–33.
3. Glazer JV, MacDonnell K, Frederick C, Ingersoll K, Ritterband LM. Liar! Liar! Identifying eligibility fraud by applicants in digital health research. Internet Interv. 2021;25:100401.
